# Supplementary material for: Pt-O bond as an active site superior to Pt0 in hydrogen evolution reaction
Source: Nat Commun. 2020 Jan 24;11:490. doi: 10.1038/s41467-019-14274-z (PMC6981163; doi:10.1038/s41467-019-14274-z)
Supplement: Supplementary file 1 — Supplementary Information [file 41467_2019_14274_MOESM1_ESM.pdf]

Supplementary information for

Pt-O bond as an active site superior to Pt^0 in hydrogen evolution reaction

Yu and Lang *et al.*

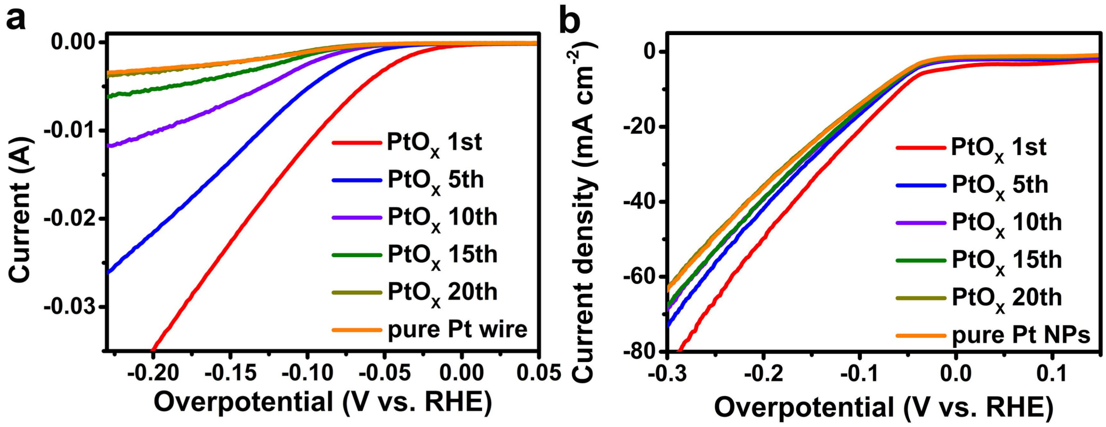

Supplementary Figure 1: HER for Pt wire and Pt nanoparticles. (a) The polarization curves of pure Pt wire and PtO_x wire. **(b)** The polarization curves of pure Pt (100) nanoparticles.

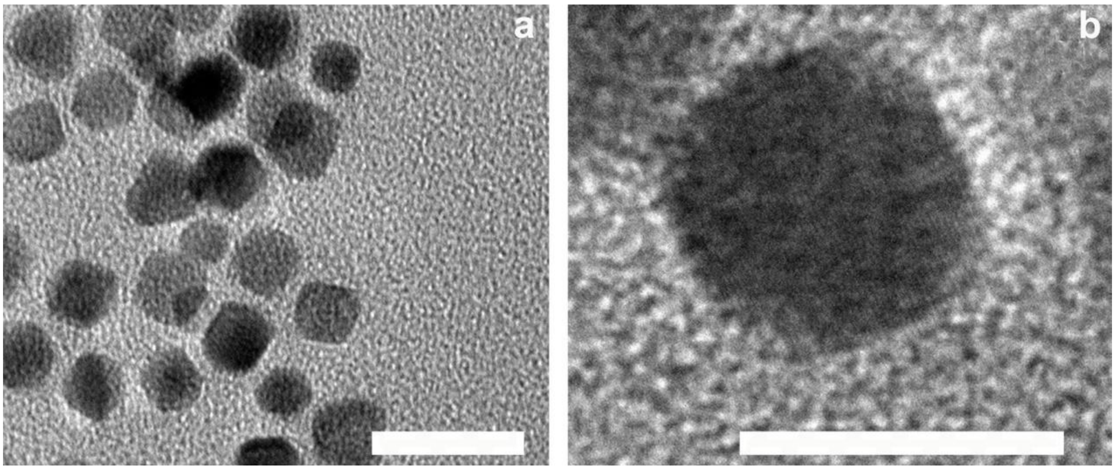

Supplementary Figure 2: TEM of Pt nanoparticles. TEM image of **(a)** cubes particles of Pt(100), **(b)** HRTEM image of a cube along the [100] zone axis. The scale bar is 20.0 nm. These results demonstrate the Pt(100) nanoparticles have been synthesized successfully.

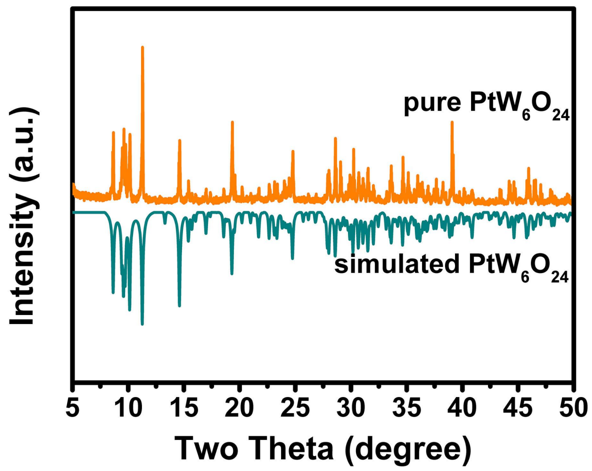

Supplementary Figure 3: XRD for PtW_6O_{24}. XRD pattern of pure PtW_6O_{24} and simulated PtW_6O_{24}. The XRD pattern obtained from the experiment is similar to that of the simulated spectrum, which proves that the obtained crystal is pure phase.

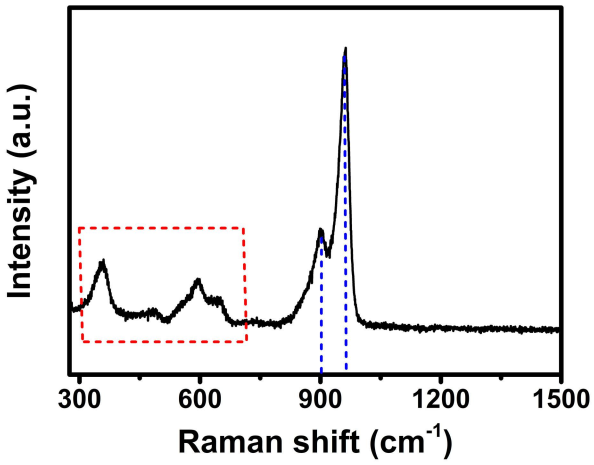

Supplementary Figure 4: Raman for PtW_6O_{24}. Raman spectroscopy of PtW_6O_{24}. Raman spectra indicates that red dotted zone means the existence of Pt-O bond.

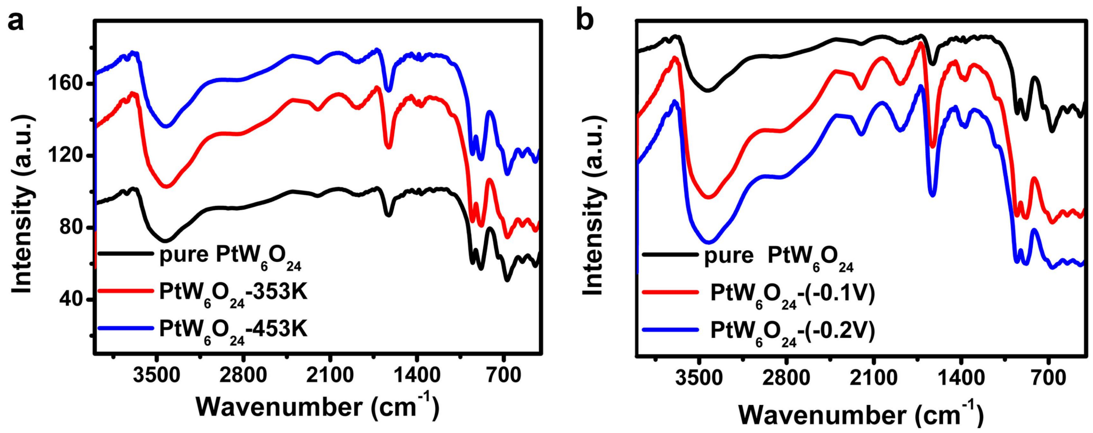

Supplementary Figure 5: IR curves of PtW_6O_{24}. (a) The IR curves of PtW_6O_{24} at different temperature for 24 h. **(b)** The IR curves of PtW_6O_{24} at different overpotential for 2 h.

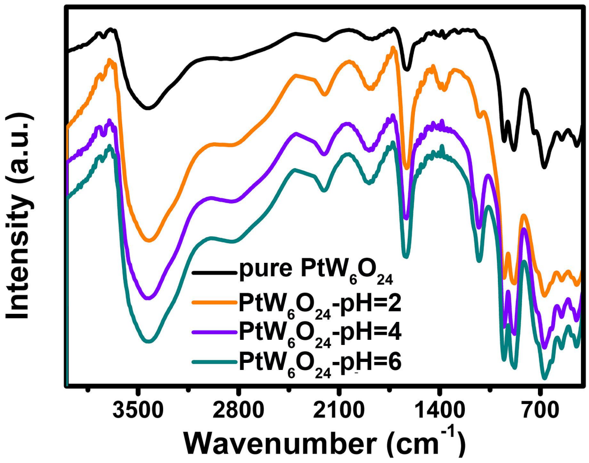

Supplementary Figure 6: IR curves of PtW_6O_{24} with different pH. The IR curves of PtW_6O_{24} at different pH for 24 h.

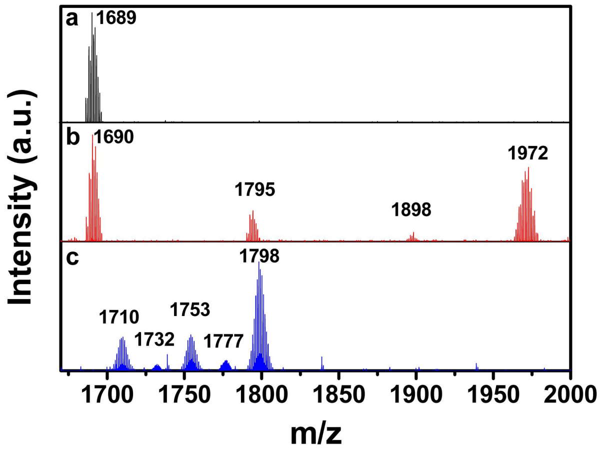

Supplementary Figure 7: The electrospray-ionization mass spectra (EIS) of PtW_6O_{24}. The electrospray-ionization mass spectra (EIS) of PtW_6O_{24} compound before and after HER in 0.5 M H_2SO_4 aqueous solution as well as its re-oxidized species by Br_2. The test samples are prepared by precipitating the polyoxoanion with tetrabutylammonium (TBA) bromide and dissolved in CH_3CN. (a) Before HER, the signals of m/z = 1689 can be assigned to $H_4[Pt^{IV}W_6O_{24}H_3]^-$ species. (b) After PtW_6O_{24} catalyst has undergone HER for 10 cycles in 0.5 M H_2SO_4 aqueous solution, the signals of m/z = 1690, m/z = 1795, m/z = 1898, and m/z = 1972 can be assigned to $H_4[Pt^{II}W_6O_{24}H_5]^-$, $NaH_3[Pt^{II}W_6O_{24}H_5]^-\cdot(CH_3CN)_2$, $Na_4[Pt^{II}W_6O_{24}H_5]^-\cdot(CH_3CN)_2(H_2O)_2$, and $(TBA)NaH_2[Pt^{II}W_6O_{24}H_5]^-\cdot CH_3CN$ species, respectively. (c) When the molecular catalyst was re-oxidized by Br_2 in above solution, the signals of m/z = 1710, m/z = 1732, m/z = 1753, m/z = 1777 and m/z = 1798 can be assigned to $NaH_3[Pt^{IV}W_6O_{24}H_3]^-$, $Na_2H_2[Pt^{IV}W_6O_{24}H_3]^-$, $NaH_3[Pt^{IV}W_6O_{24}H_3]^-\cdot CH_3CN$, $Na_4[Pt^{IV}W_6O_{24}H_3]^-$, and $Na_3H[Pt^{IV}W_6O_{24}H_3]^-\cdot CH_3CN$, respectively. The above experiments demonstrate that PtW_6O_{24} compound behaves as a reversible redox-active catalyst.

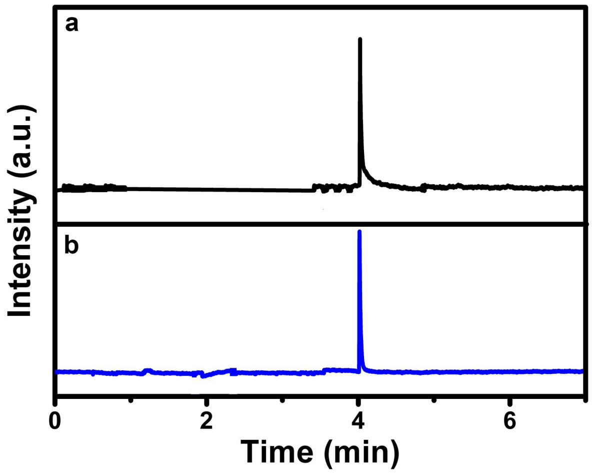

Supplementary Figure 8: The CE of PtW_6O_{24} before and after HER. Electropherogram for 0.25 mM of PtW_6O_{24} compound in a 20 mM NaH_2PO_4-H_3PO_4 buffer (pH=3) before HER (a), and its re-oxidized species by Br_2 after HER (b). The retention time of PtW_6O_{24} before HER and its re-oxidized species by Br_2 after HER almost unchanged, further confirming that PtW_6O_{24} compound is a reversible redox-active catalyst.

Experimental conditions for capillary electrophoresis: Fused-silica capillaries (50 µm i.d., 365µm o.d., Hebei Yongnian Factory, China) with total length of 50 cm and effective length of 41 cm were used. The detection wavelength was set at 232 nm. The running buffer for CE separation was 20amM NaH_2PO-H_3PO_4 solution (pH=3). The separation voltage was set at -20 kV. The sample was hydrodynamically injected into the capillary (10 cm, 20 s)

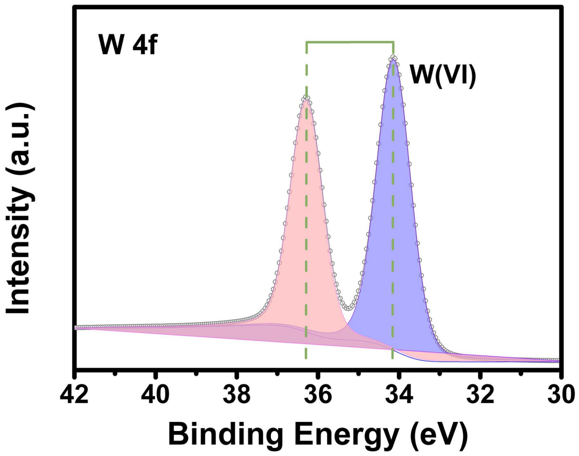

Supplementary Figure 9: The XPS spectra of W. High-resolution XPS spectra of W. the W $4f_{7/2}$ and W $4f_{5/2}$ located at 34.2 and 36.3 eV, respectively, which are consistent with the presence of W(VI) as reported in the literatures.

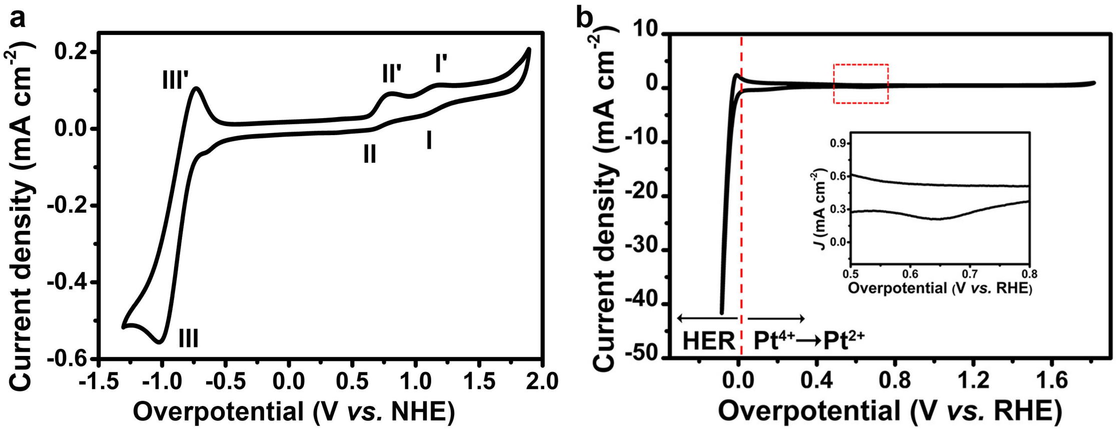

Supplementary Figure 10:The CV of PtW_6O_{24} in acetonitrile and 0.5 M H_2SO_4. **(a)** CV of PtW_6O_{24} in acetonitrile with the scan rate of 30 mV s^{-1}. **(b)** CV of PtW_6O_{24} in 0.5 M H_2SO_4 with the scan rate of 20 mV s^{-1}.

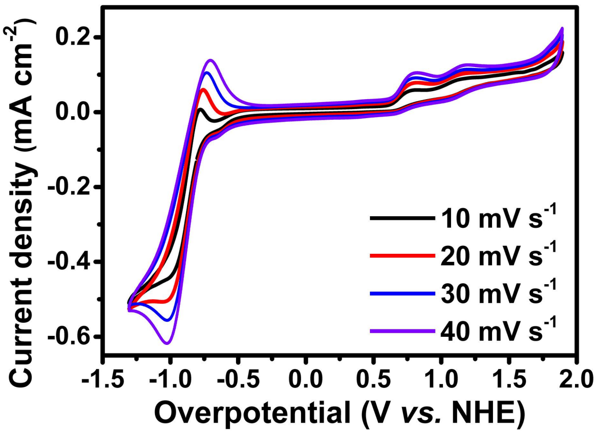

Supplementary Figure 11: The CV of PtW_6O_{24} in acetonitrile. The cyclic voltammetry of PtW_6O_{24} in acetonitrile with different scan rates.

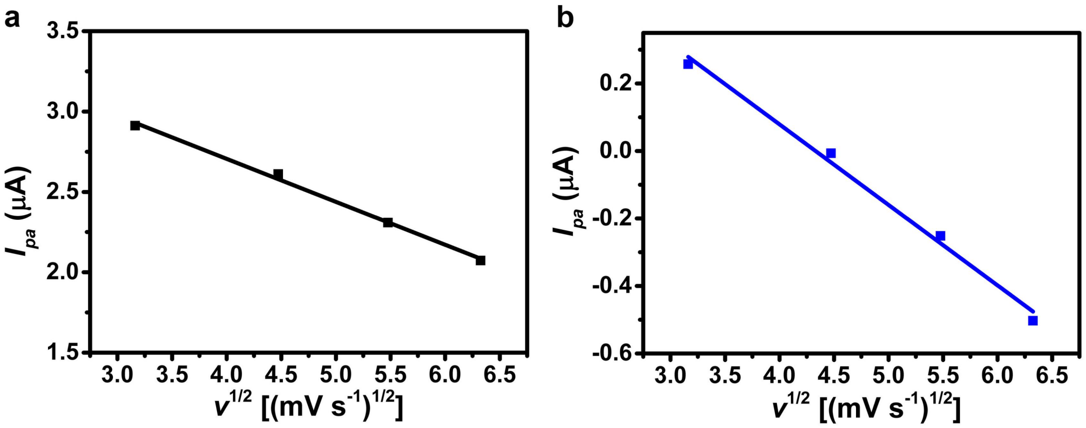

Supplementary Figure 12: The linear relationship between I_{pa} and $v^{1/2}$. (a) The graph of linear relationship between I_{pa} and $v^{1/2}$ of the first reduction wave. **(b)** The graph of linear relationship between I_{pa} and $v^{1/2}$ of the second reduction wave.

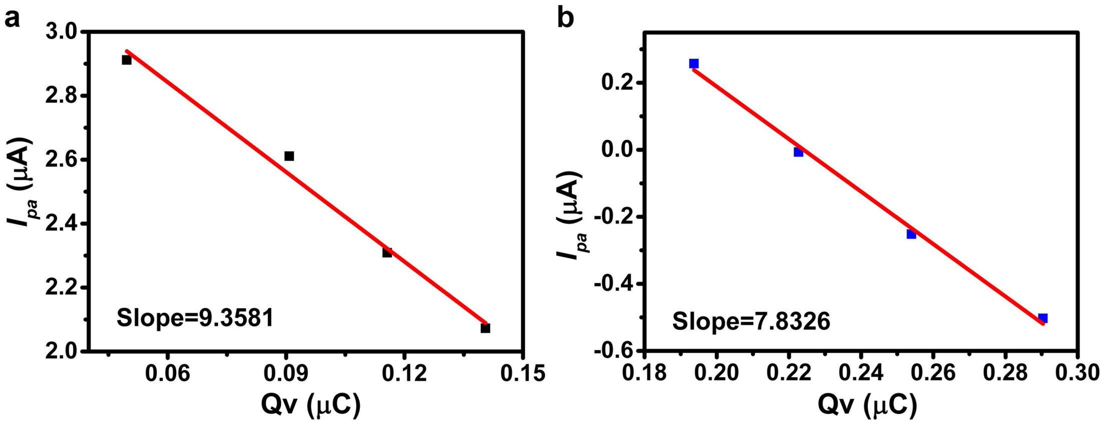

Supplementary Figure 13: The linear relationship of Q_v and I_{pa}. (a) The graph of linear relationship of Q_v and I_{pa} of the first reduction wave. **(b)** The graph of linear relationship of Q_v and I_{pa} of the second reduction wave.

When I_p is linear with $v^{1/2}$, the adsorption of the substance on the electrode conforms to the Langumir adsorption isotherm.

$$I_p=n^2F^2vA\Gamma_T/4RT=nFQ_v/4RT. \quad \text{(Supplementary Equation 1)}$$

The number of reduced electrons of Pt in PtW_6O_{24} can be further calculated by the Langmuir adsorption isotherm. During the PtW_6O_{24} redox process, there are only two one-electron reduction process of Pt, which further indicated that there is absence of metallic Pt.

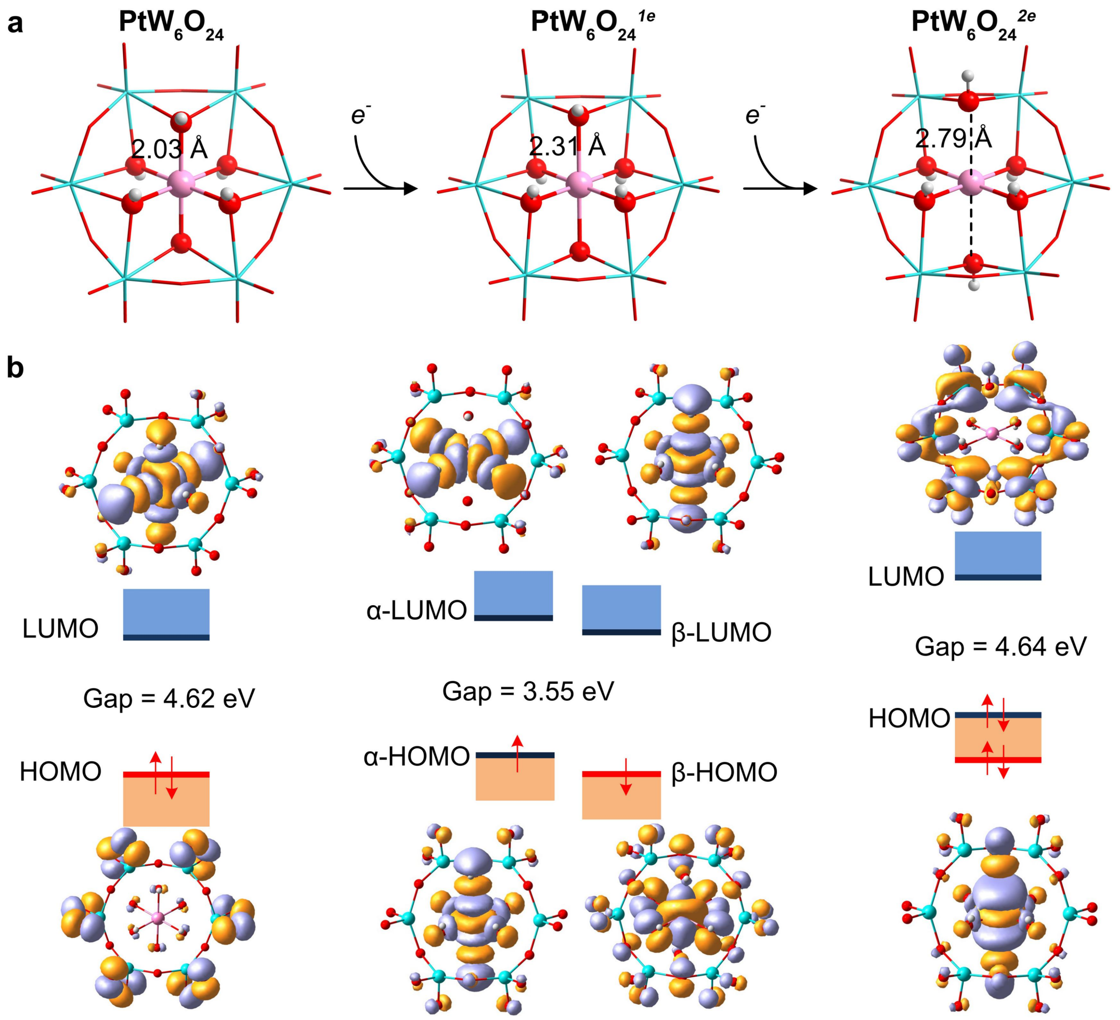

Supplementary Figure 14: Geometric changes for $H_6PtW_6O_{24}$ based on the continuous reduction processes. **(a) The structure of PtW_6O_{24}.** (b) 3D representation of the highest occupied molecular orbital (HOMO) and the lowest unoccupied molecular orbital (LUMO) for $H_6PtW_6O_{24}$, $[H_6PtW_6O_{24}]^{1e}$, and $[H_6PtW_6O_{24}]^{2e}$.

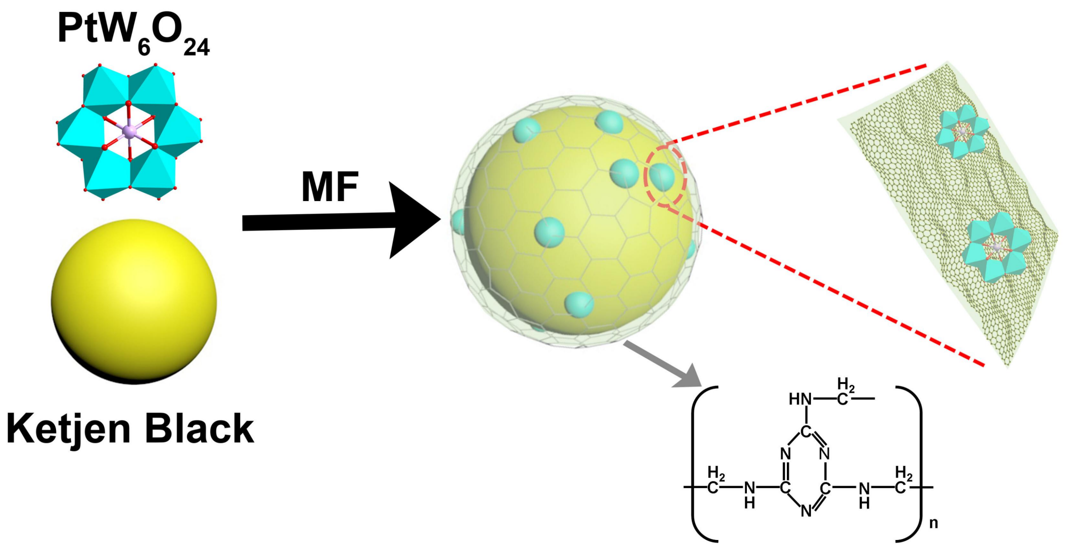

Supplementary Figure 15: Scheme illustration of the synthetic process of PtW_6O_{24}/C. First, 0.065 g the crystal of $Na_5[H_3PtW_6O_{24}]$ (PtW_6O_{24}) was uniformly dispersed in 1 mL aqueous solution. Subsequently, 5 mg Ketjen black carbon was added to and stirred at room temperature for 2 hours to ensure PtW_6O_{24} and Ketjen black carbon can be uniformly distributed in the aqueous solution. Then, 10 μL Melamine-Formaldehyde (MF) was added to as an adhesive to bond the crystal to Ketjen black carbon. At the same time, MF will replace the counter cations in the crystal to stabilize the crystal on the Ketjen black carbon. After stirring at room temperature for 4 hours, the electrocatalyst can be obtained by centrifugation and dried by vacuum. $Na_5[H_3PtW_6O_{24}]$, an effective component of electrocatalysts, is helpful to further observe the electrocatalytic process at the molecular level because of its identified structure. As a superconducting carbon material, Ketjen black carbon not only be the substrate to disperse PtW_6O_{24} molecules, but also provide good conductivity for electron transfer. We can prepare a series of electrocatalysts with different loading of PtW_6O_{24} by adjusting the mass of PtW_6O_{24}.

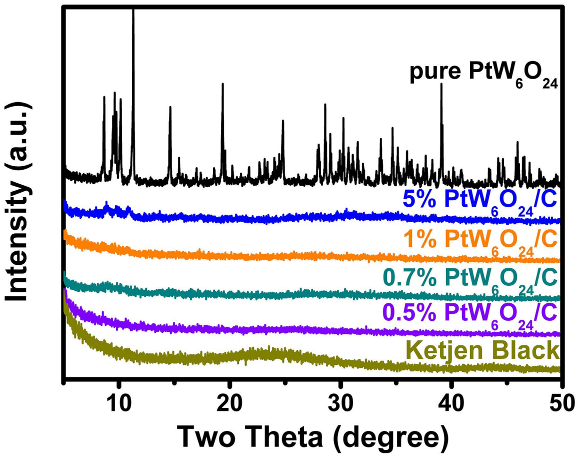

Supplementary Figure 16: The XRD pattern of different content of PtW_6O_{24}/C. The broad peak at 25 degree can be attributed to Ketjen black carbon. There are no peaks on PtW_6O_{24}/C implies that the size of PtW_6O_{24} species in PtW_6O_{24}/C is below the detection limit, possibly in the monodisperse regime.

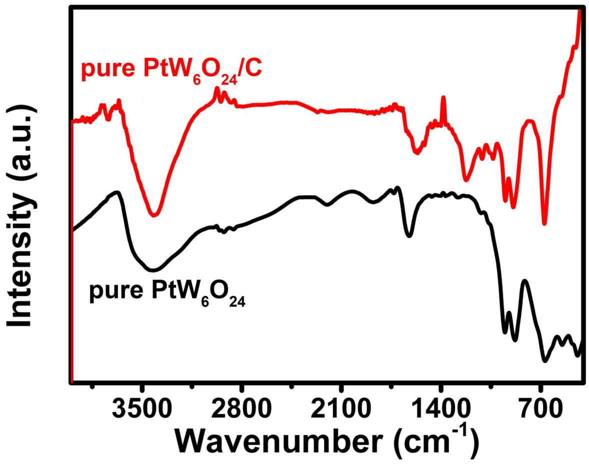

Supplementary Figure 17: Infrared Spectroscopy (IR) of pure PtW_6O_{24} and PtW_6O_{24}/C. The IR of pure PtW_6O_{24} is similar with that of PtW_6O_{24}/C, which proves that PtW_6O_{24}/C remain its structure before and after loading on carbon.

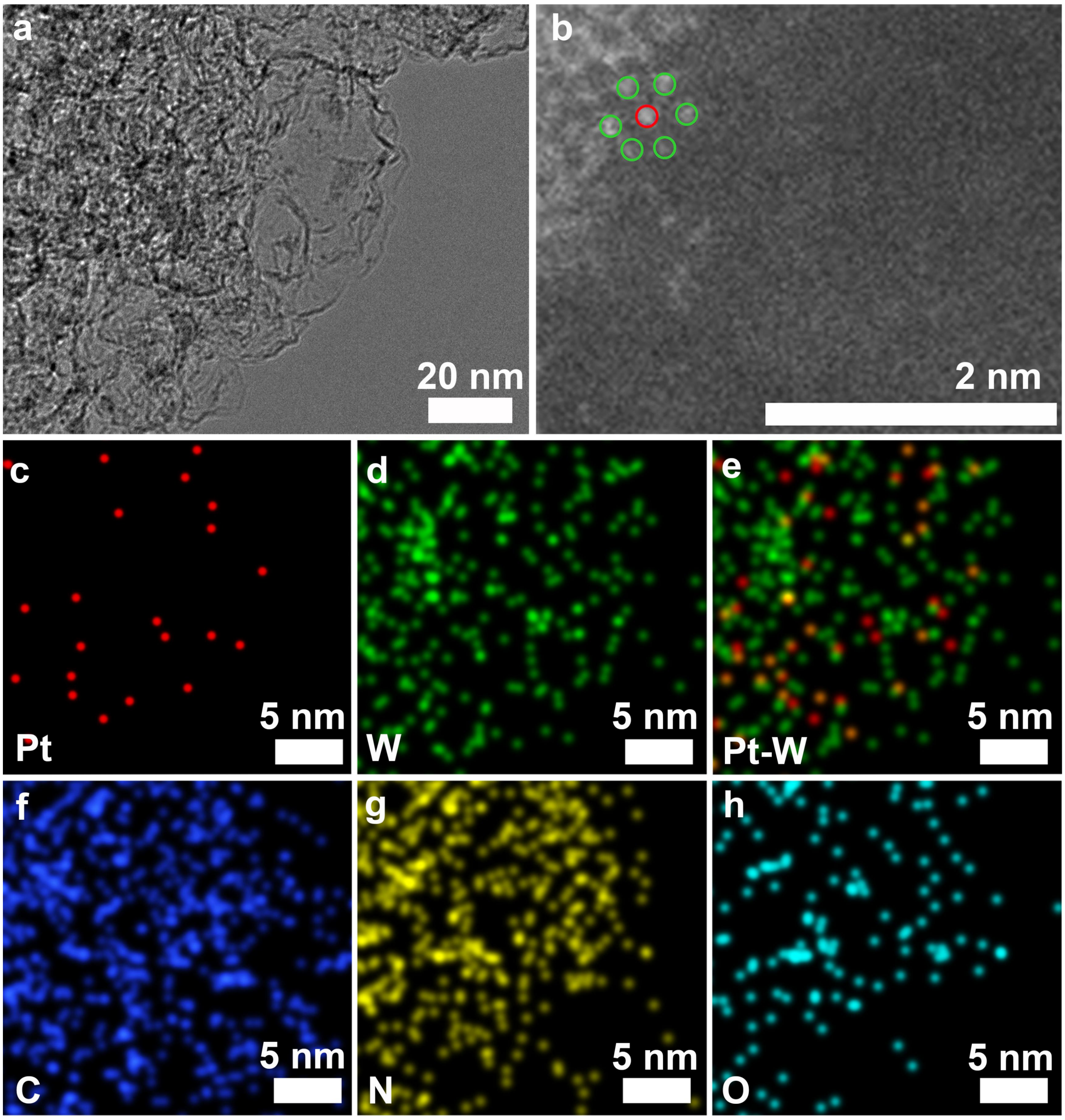

Supplementary Figure 18: TEM of PtW_6O_{24}/C. **(a)** TEM of PtW_6O_{24}/C. **(b)** STEM of PtW_6O_{24}/C. **(c-g)** Element mapping of PtW_6O_{24}/C of, Pt **(c)**, W **(d)**, Pt-W **(e),** C **(f),** N **(g)** and C **(g)**.

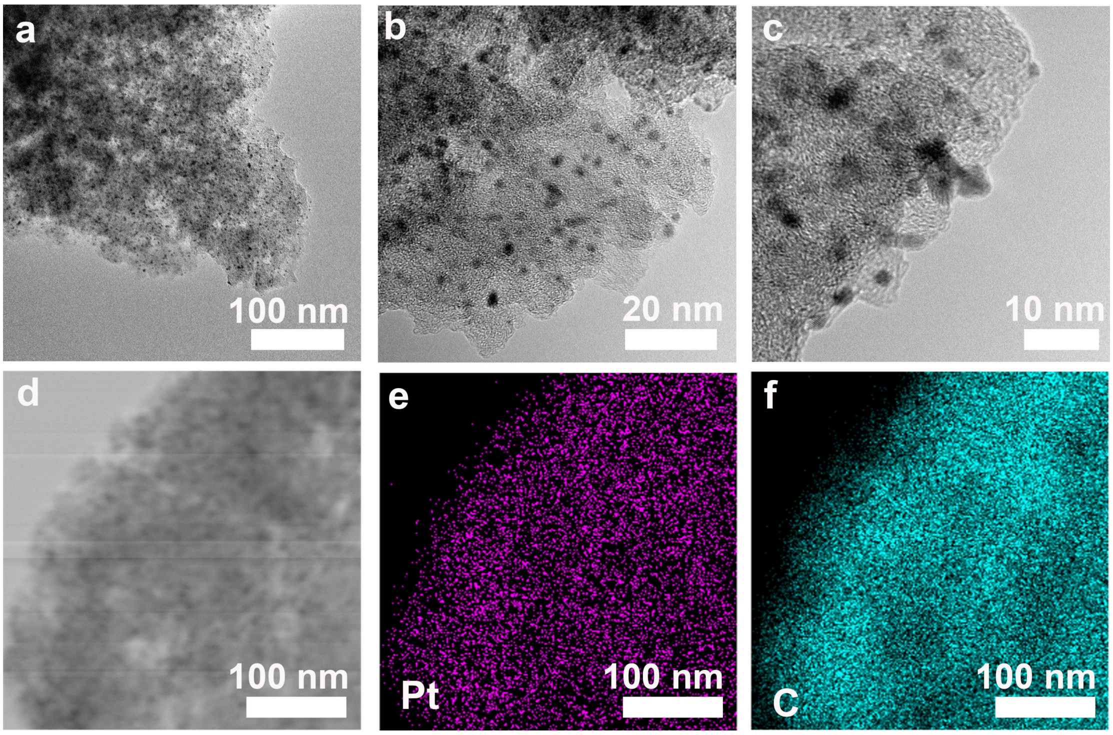

Supplementary Figure 19: TEM of 20% Pt/C. (a) TEM of 20% Pt/C. **(b)** HRTEM of 20% Pt/C. **(d-f)** Element mapping of 20% Pt/C Pt **(c)**, W **(d)**, Pt-W **(e), ** C **(f),** N **(g)** and C **(g).**

Supplementary Figures 18-19 shows homogeneous dispersion of crystal PtW_6O_{24} on Ketjen black, and no Pt NPs or clusters were observed, possibly meaning the PtW_6O_{24} in the monodisperse regime, which is obviously different with 20% Pt/C. The corresponding HAADF image displays isolated bright dots, which can be attributed to the heavy Pt and W atoms, further indicating PtW_6O_{24} molecules were monodisperse. The elemental mapping verifies the presence of Pt, W, O and C elements. The above results demonstrate crystal PtW_6O_{24} molecules are successfully monodispersed on Ketjen black.

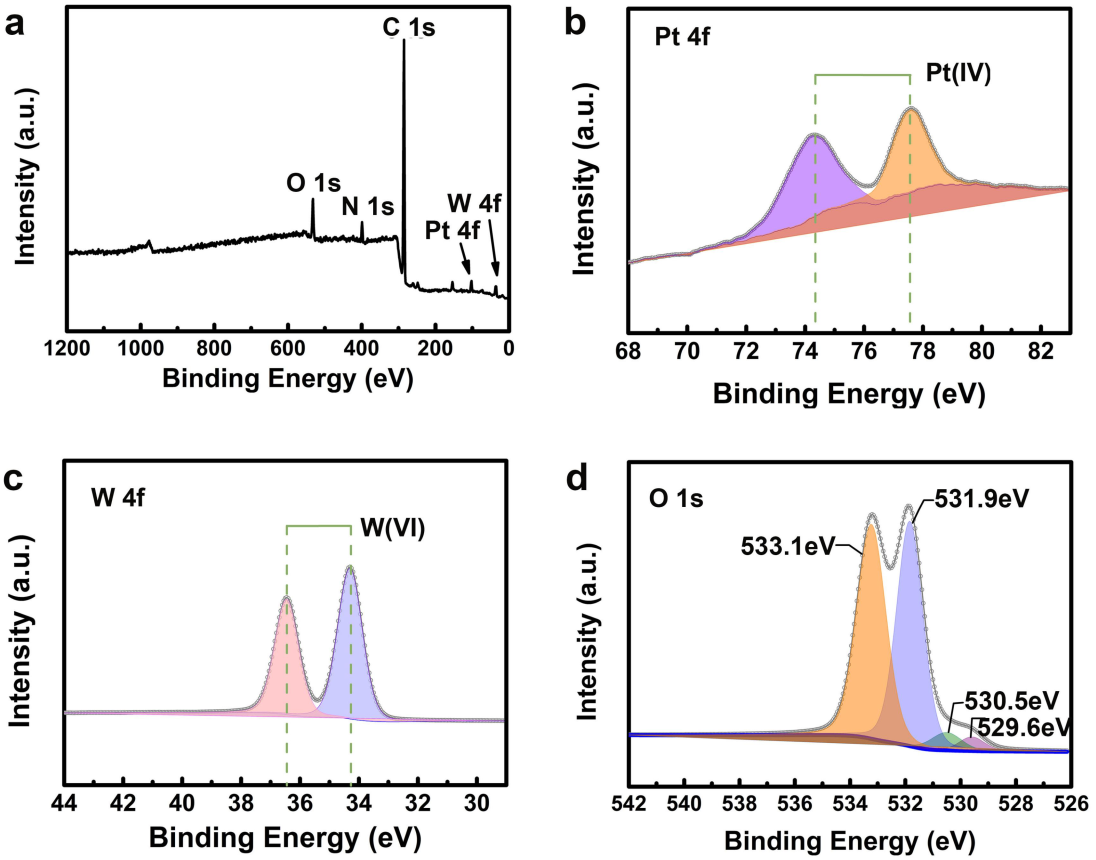

Supplementary Figure 20： XPS for PtW_6O_{24}/C. (a) The full XPS spectra for PtW_6O_{24}/C. (**(b, c, d)** High-resolution XPS spectra of Pt, W and O for PtW_6O_{24}.

Supplementary Figure 20a reveals the full XPS spectrum of PtW_6O_{24}/C, which verifies the presence of Pt, W, O and C elements. The high resolution XPS spectra of Pt in Supplementary Figure 20b, the Pt $4f_{7/2}$ and Pt $4f_{5/2}$ located at 74.3 and 77.7 eV, respectively, which are consistent with the presence of Pt(IV) as reported in the literatures. It is notably that there are not existence of peak at 71.4 and 74.7 eV, implying the absence of metallic Pt. The W XPS spectra with two peaks at 34.4 eV and 36.6 eV assigned to $4f_{7/2}$ and $4f_{5/2}$ of W(VI) (Supplementary Figure 20c). The XPS spectra of O 1s is shown in Supplementary Figure 20d, the peak at 529.6 eV can be attributed to W=O. The signal at 530.5 eV is related to the W-O-W bond. The peak at 531.9 eV is assigned to oxygen present on the surface of carbon, which may come from oxygen adsorbed on carbon, and covers the peak of Pt-O bond. The peak at 533.1 eV is distribute to the presence of the hydroxyl (O-H), which may be attributed to the presence of hydrogen protons attached to oxygen atoms and the crystal H_2O adsorbed throughout the PtW_6O_{24} molecule. All little shifts of peaks are ascribed to the interaction of PtW_6O_{24} with carbon.

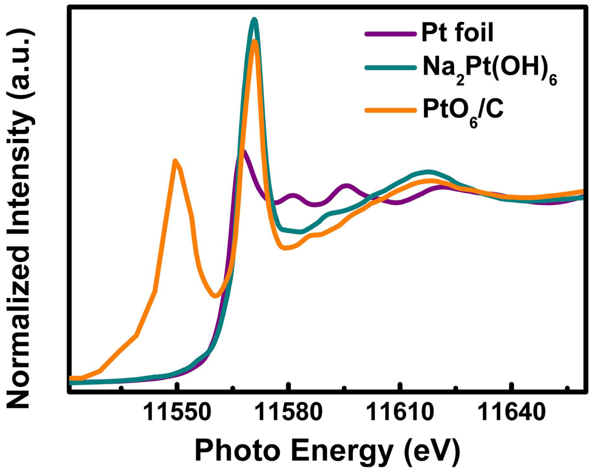

Supplementary Figure 21: Pt *L*$_3$-edge XANES spectra of PtW_6O_{24}/C, $Na_2Pt(OH)_6$ and Pt foil. Feature B is due to the interference of W *L*$_2$-edge, which is not affect the analysis of main peak of PtW_6O_{24}/C.

The normalized XANES spectra of PtW_6O_{24}/C and reference materials ($Na_2Pt(OH)_6$ and Pt foil) was shown in Supplementary Figure 21. The Pt white-line intensity for PtW_6O_{24}/C is similar to that of $Na_2Pt(OH)_6$, which means the valence state of Pt remained unchanged during the preparation of catalyst, which is consistent with the results of XPS.

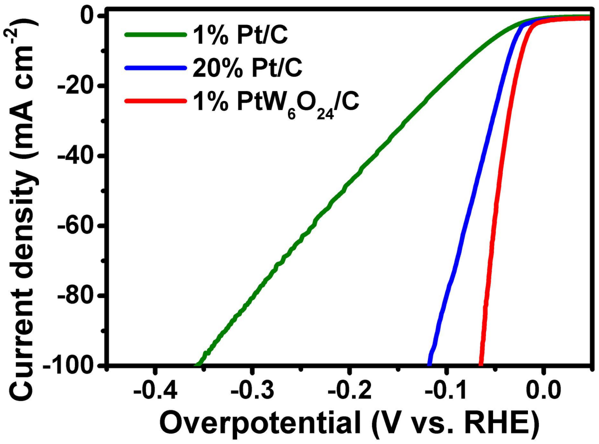

Supplementary Figure 22: LSV of 1% PtW_6O_{24}/C and 20% Pt/C and 1% Pt/C. The polarization curves of 1% PtW_6O_{24}/C and 20% Pt/C at the current density 10 mA cm^{-2} in N_2-saturated 0.5 M H_2SO_4.

Series of PtW_6O_{24}/C with different loading of Pt have been prepared. As described in Supplementary Figure 22, with the increase of Pt loading, the HER performance of catalysts was obviously enhanced. When the loading of Pt increase to 5%, PtW_6O_{24} are aggregated, resulting in a decrease of catalyst utilization and a slight enhancement in HER performance which is compared with 1% PtW_6O_{24}/C.

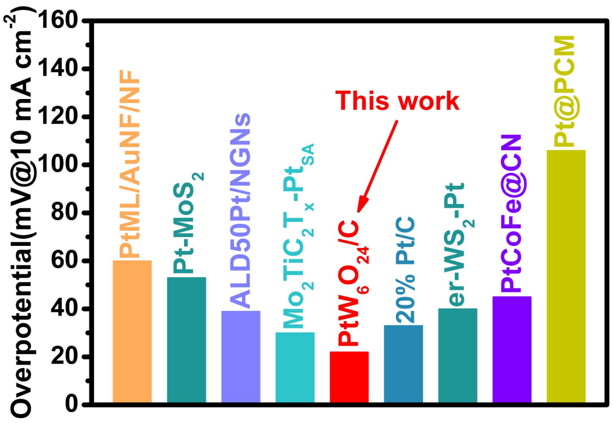

Supplementary Figure 23: Comparison of PtW_6O_{24}/C and reported Pt-based electrocatalysts. Comparison of overpotentials of PtW_6O_{24}/C and reported Pt-based electrocatalysts at 10 mA cm^{-2}.

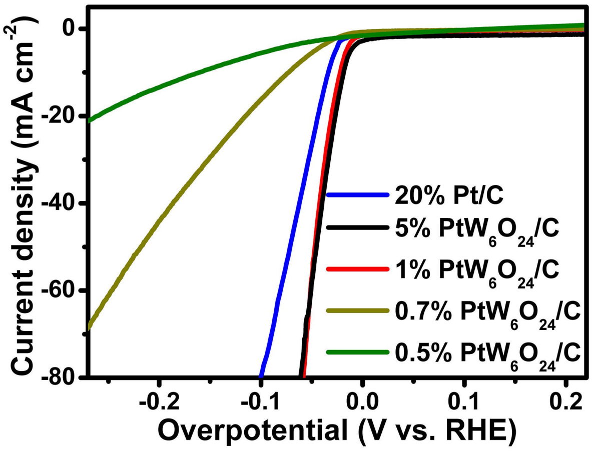

Supplementary Figure 24: LSV of PtW_6O_{24}/C with different Pt content The HER polarization curves of PtW_6O_{24}/C in 0.5 M H_2SO_4 at rate of 5 mV s^{-1}.

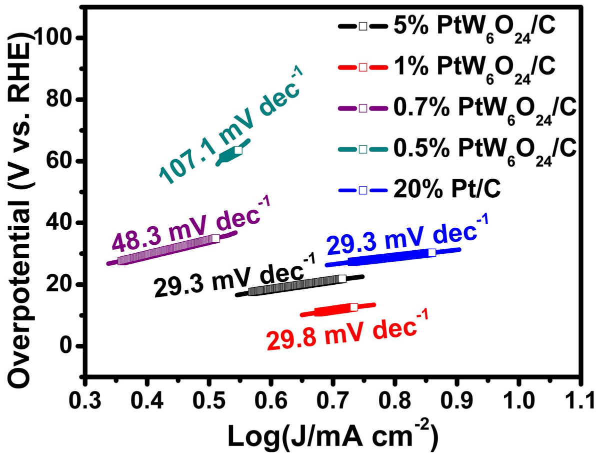

Supplementary Figure 25: Tafel slope of PtW_6O_{24}/C. Tafel slope of PtW_6O_{24}/C with different amounts of PtW_6O_{24}/C under the optimal conditions.

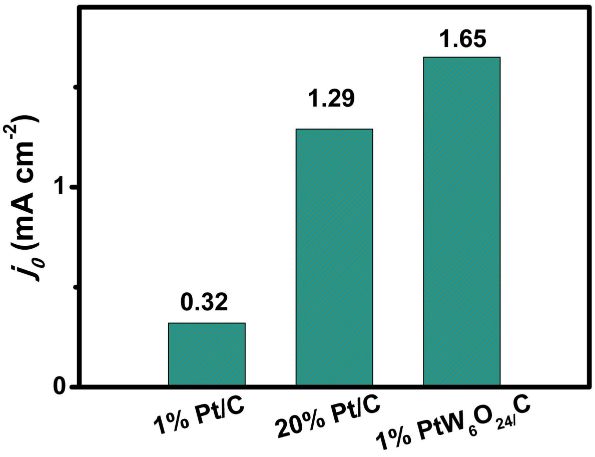

Supplementary Figure 26: The exchange current density of PtW_6O_{24}/C. The exchange current density of 1% PtW_6O_{24}/C, 20% Pt/C and 1% Pt/C.

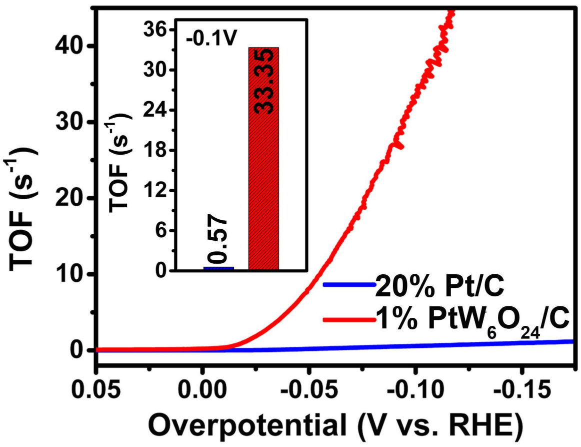

Supplementary Figure 27: TOF of PtW_6O_{24}/C. TOF of 1% PtW_6O_{24}/C and 20% Pt/C.

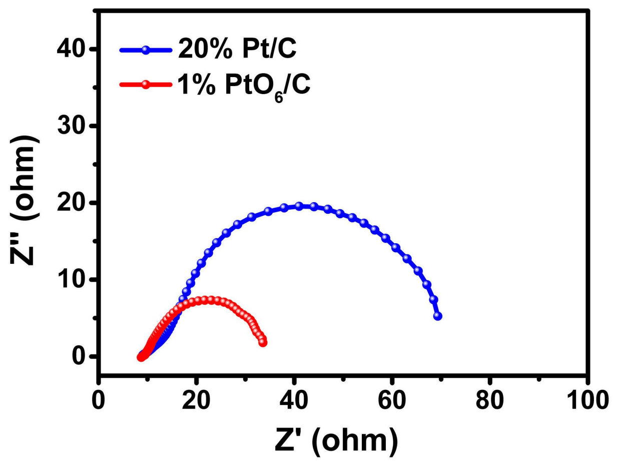

Supplementary Figure 28: The Nyquist plot of PtW_6O_{24}/C. The Nyquist plot of 1% PtW_6O_{24}/C and 20% Pt/C at the overpotential of 40 mV vs RHE.

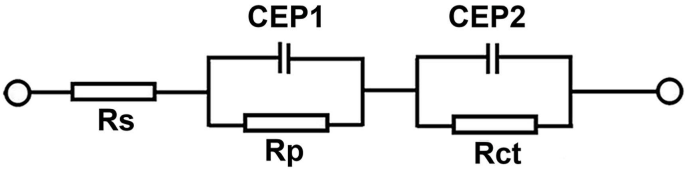

Supplementary Figure 29: The equivalent circuit model. Two-time-constant model equivalent circuit used for data fitting of EIS spectra (R_s represents the overall series resistance, CPE1 and CPE2 represent the constant phase element and resistance related to surface porosity R_p, and R_{ct} represents the charge transfer resistance related to HER process).

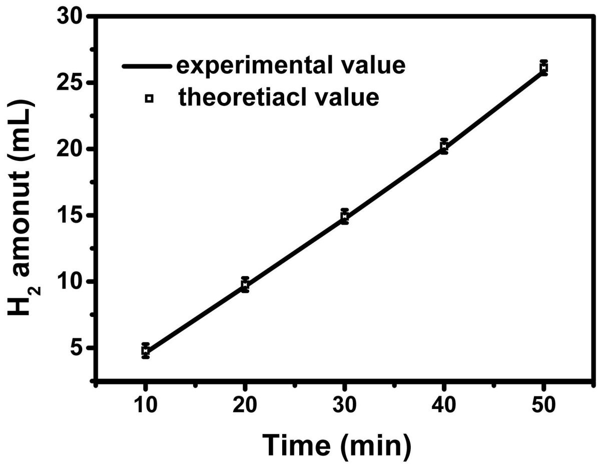

Supplementary Figure 30: The FE of PtW_6O_{24}/C. The Faradic efficiency of 1% PtW_6O_{24}/C.

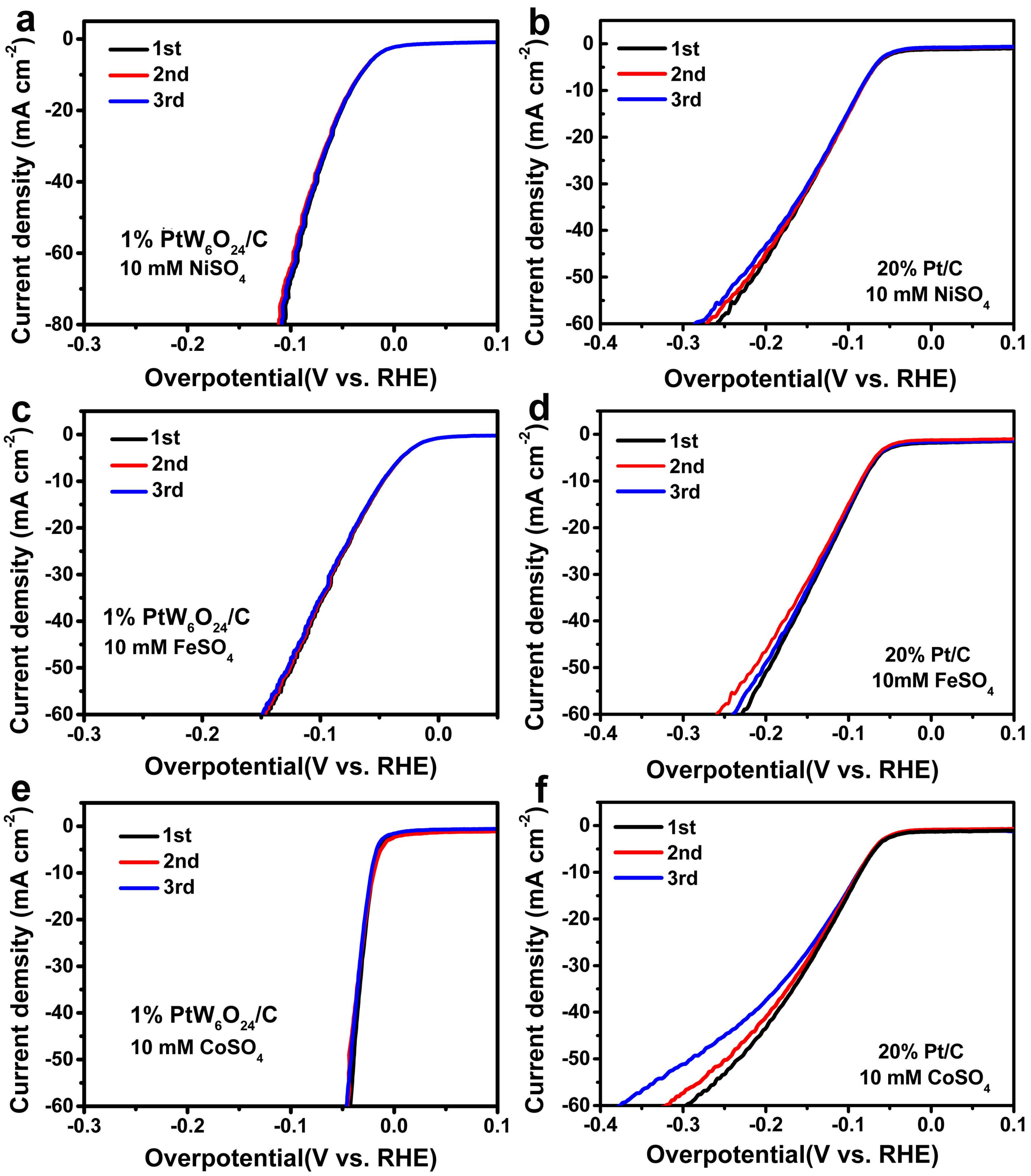

Supplementary Figure 31: Anti-toxicity test of PtW_6O_{24}/C and 20% Pt/C. (a) and **(b)** The HER polarization curves of 1% PtW_6O_{24}/C and 20% Pt/C in 10 mM $MnSO_4$. **(c)** and **(d)** The HER polarization curves of 1% PtW_6O_{24}/C and 20% Pt/C in 10 mM $NiSO_4$. **(e)** and **(f)** The HER polarization curves of 1% PtW_6O_{24}/C and 20% Pt/C in 10 mM $FeSO_4$.

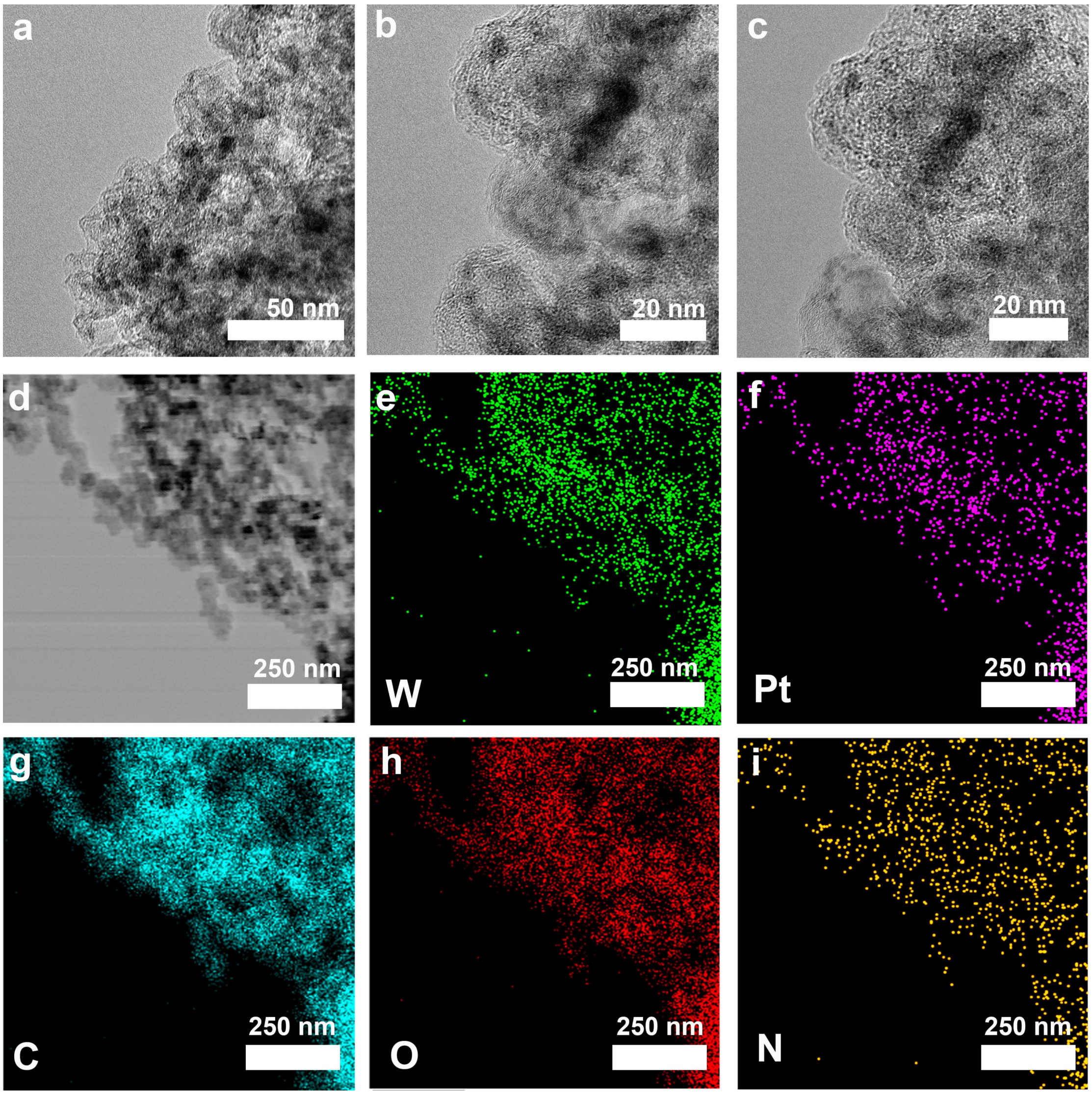

Supplementary Figure 32: TEM of PtW_6O_{24}/C after HER. (a) TEM images of 1% PtW_6O_{24}/C after electrochemical tests. **(b-c)** HRTEM images of 1% PtW_6O_{24}/C after electrochemical tests. **(d-i)** Elemental mapping of 1% PtW_6O_{24}/C after electrochemical tests.

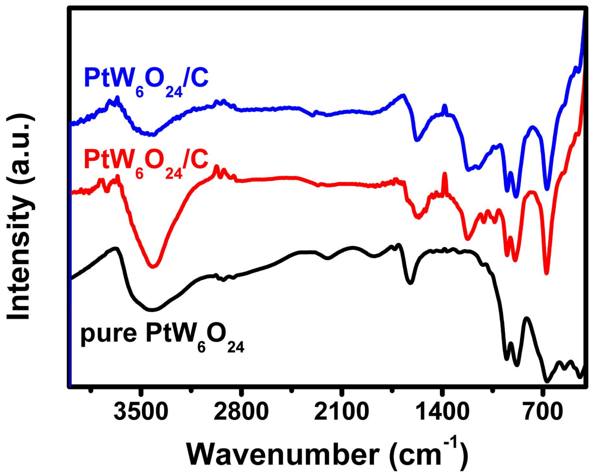

Supplementary Figure 33: IR spectroscopy of PtW_6O_{24}/C after HER. IR spectroscopy of pure molecule PtW_6O_{24}, 1% PtW_6O_{24}/C before electrochemical hydrogen evolution reaction and PtW_6O_{24}/C after electrochemical hydrogen evolution reaction.

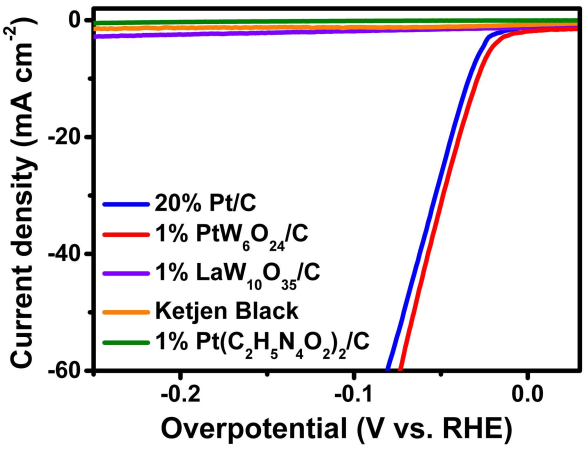

Supplementary Figure 34: LSV of PtW_6O_{24}/C and the corresponding comparisons. The polarization curves of PtW_6O_{24}/C and the corresponding comparisons in N_2-saturated 0.5 M H_2SO_4.

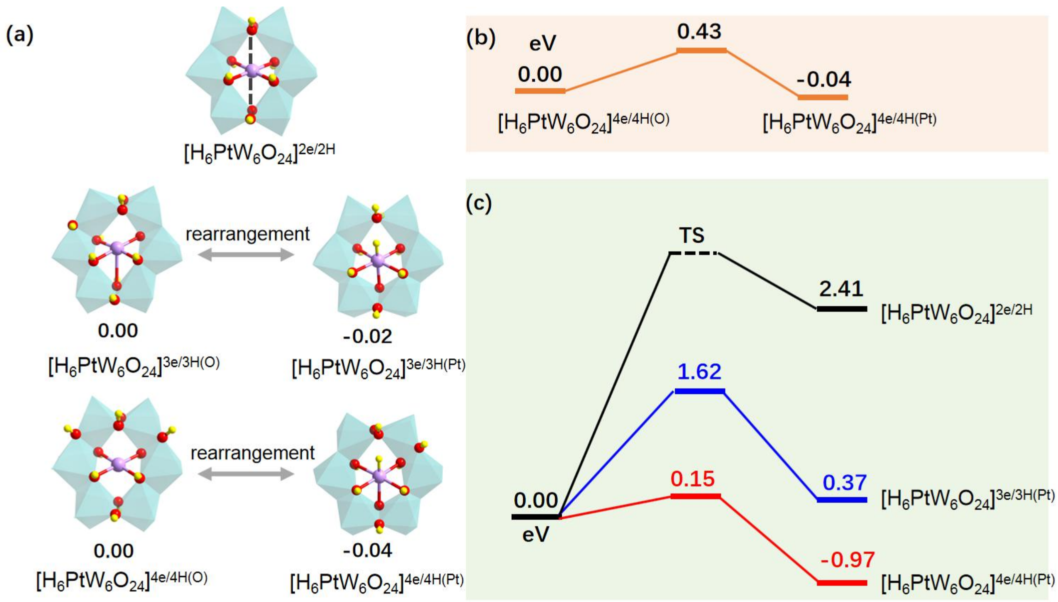

Supplementary Figure 35: Detailed H_2 evolution pathways over [$H_6PtW_6O_{24}$]. (a) The polyhedral and ball-stick representation for different reduction states, and the intramolecular electronic rearrangement process (energy in eV) for $[H_6PtW_6O_{24}]^{3e/3H}$ and $[H_6PtW_6O_{24}]^{4e/4H}$ intermediates. The [$H_6PtW_6O_{24}$] was easily reduced to $[H_6PtW_6O_{24}]^{2e}$ and accumulated two additional protons on the bridge oxygen sites to form $[H_6PtW_6O_{24}]^{2e/2H}$, and further reduced to $[H_6PtW_6O_{24}]^{3e/3H}$ and $[H_6PtW_6O_{24}]^{4e/4H}$. Mulliken charge analysis shows almost no change for charge on Pt when reduced from $[H_6PtW_6O_{24}]^{2e/2H}$ to $[H_6PtW_6O_{24}]^{3e/3H(O)}$ and $[H_6PtW_6O_{24}]^{4e/4H(O)}$ at M06/6-31G**/LANL2DZ/PCM level. On the other hand, a decrease of charge on Pt from 0.74 to 0.46 and 0.55 was obtained when the $[H_6PtW_6O_{24}]^{3e/3H(O)}$ and $[H_6PtW_6O_{24}]^{4e/4H(O)}$ rearranged to $[H_6PtW_6O_{24}]^{3e/3H(Pt)}$ and $[H_6PtW_6O_{24}]^{4e/4H(Pt)}$ respectively. This indicates the adsorption of H on Pt has led to the reorganization of electronic configuration, by transferring one electron from W to PtO_4 center. It is worth noting in $[H_6PtW_6O_{24}]^{3e/3H(Pt)}$ and $[H_6PtW_6O_{24}]^{4e/4H(Pt)}$ the transferred electrons are delocalized over the whole PtO_4 moiety (Supplementary Figure 34) instead of the absolute Pt center, thus the oxidation state of Pt is larger than +1 and less than +2. **(b)** Free energy potential for reorganization between $[H_6PtW_6O_{24}]^{4e/4H(O)}$ and $[H_6PtW_6O_{24}]^{4e/4H(Pt)}$. Only 0.43 eV barrier is required, which is easily to occur under catalytic condition. Here one water molecular was involved in the model for mediating the proton transfer. (c) Free energy diagrams for H_2 production. The pathway from $[H_6PtW_6O_{24}]^{2e/2H}$ is ruled out because of the high energy demand (2.41 eV). Although the H_2 generation from $[H_6PtW_6O_{24}]^{3e/3H(Pt)}$ is little endothermic with value of 0.37 eV, such a step was computed to experience a significantly high barrier of 1.62 eV. Starting from the high reduced $[H_6PtW_6O_{24}]^{4e/4H}$, the configuration with one H adsorbed on Pt is found 0.04 eV more favorable than on the oxygen. And H_2 production is thermodynamically downhill by 0.97 eV with barrier of only 0.15 eV. This extremely low barrier is consistent with the excellent HER performance of $H_6PtW_6O_{24}$ in experiment.

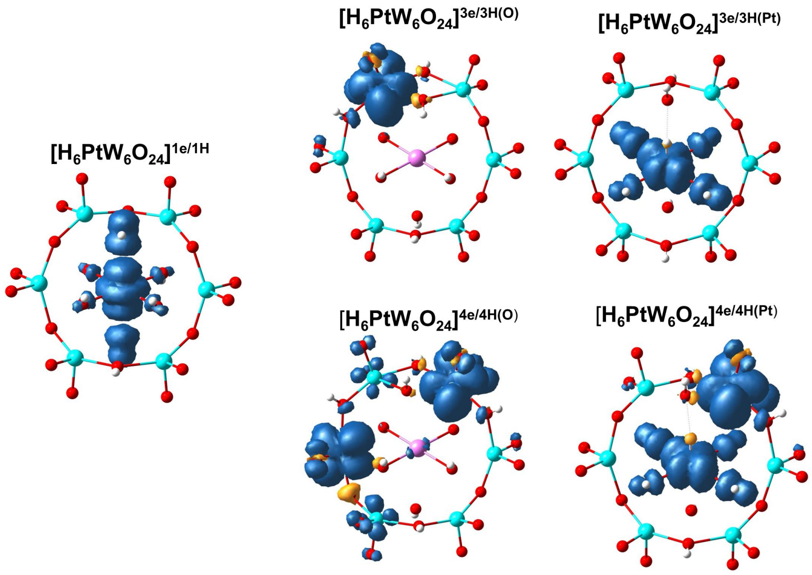

Supplementary Figure 36: The spin density representation for different reduced species of $[H_6PtW_6O_{24}]$. $[H_6PtW_6O_{24}]^{1e/1H}$ (doublet), $[H_6PtW_6O_{24}]^{3e/3H(O)}$ (doublet), $[H_6PtW_6O_{24}]^{3e/3H(Pt)}$ (doublet), $[H_6PtW_6O_{24}]^{4e/4H(O)}$ (triplet), $[H_6PtW_6O_{24}]^{4e/4H(Pt)}$ (triplet) respectively. The first reduced electron is delocalized over the PtO_6 moiety, while the third and fourth electrons are delocalized over WO moiety. It is worth noting in $[H_6PtW_6O_{24}]^{3e/3H}$ and $[H_6PtW_6O_{24}]^{4e/4H}$, configuration reorganization occurs by transferring one proton and electron, the transferred electron was delocalized over the whole PtO_4 moiety instead of the absolute Pt center, thus the oxidation state of Pt is actually larger than +1 and less than +2.

Characterization and HER performance of $Pt_2(W_5O_{18})_2$/C

Supplementary note 1

We further explore characterization of electrocatalyst $Pt_2(W_5O_{18})_2$/C shown in Supplementary Figure 37-42. Raman spectra indicates that red dotted zone means the existence of Pt-O bond (Supplementary Figure 37). The XPS spectra are shown in Supplementary Figure 40. The Pt $4f_{7/2}$ and Pt $4f_{5/2}$ located at 74.3 and 77.7 eV, respectively, which are consistent with the values of Pt^{4+} as reported in the literatures and no presence of metallic Pt. The normalized XANES spectra implies the existence of Pt(IV) (Supplementary Figure 41-42).

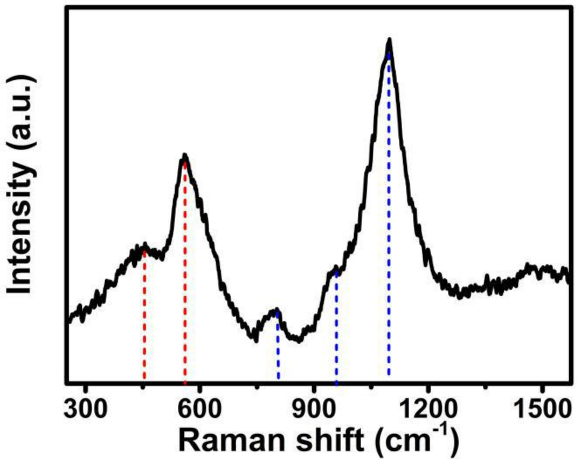

Supplementary Figure 37: Raman spectroscopy of $Pt_2(W_5O_{18})_2$. Raman spectra indicates that red dotted zone means the existence of Pt-O bond. The peak positions of 441 and 562 cm^{-1} may be ascribed to the Pt-O vibration, while peak positions of 806, 955 and 1098 cm^{-1} could be attributed to W-O vibration.

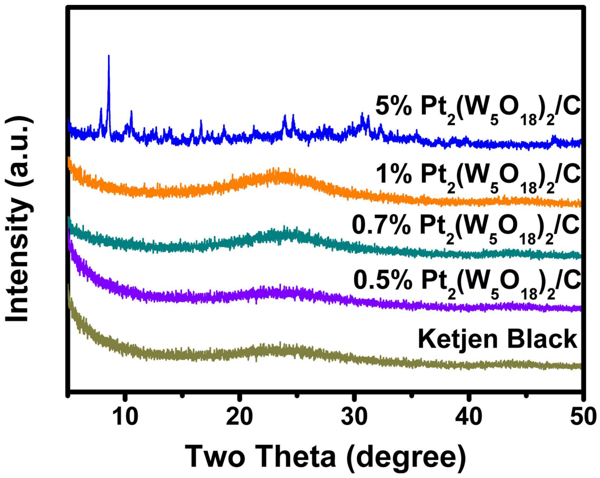

Supplementary Figure 38: The XRD pattern of different content of $Pt_2(W_5O_{18})_2/C$. The broad peak at 25 degree can be attributed to ketjen black. There are on peaks on $Pt_2(W_5O_{18})_2/C$ implies that the size of $Pt_2(W_5O_{18})_2/C$ species in $Pt_2(W_5O_{18})_2/C$ is below the detection limit, possibly in the monodisperse regime.

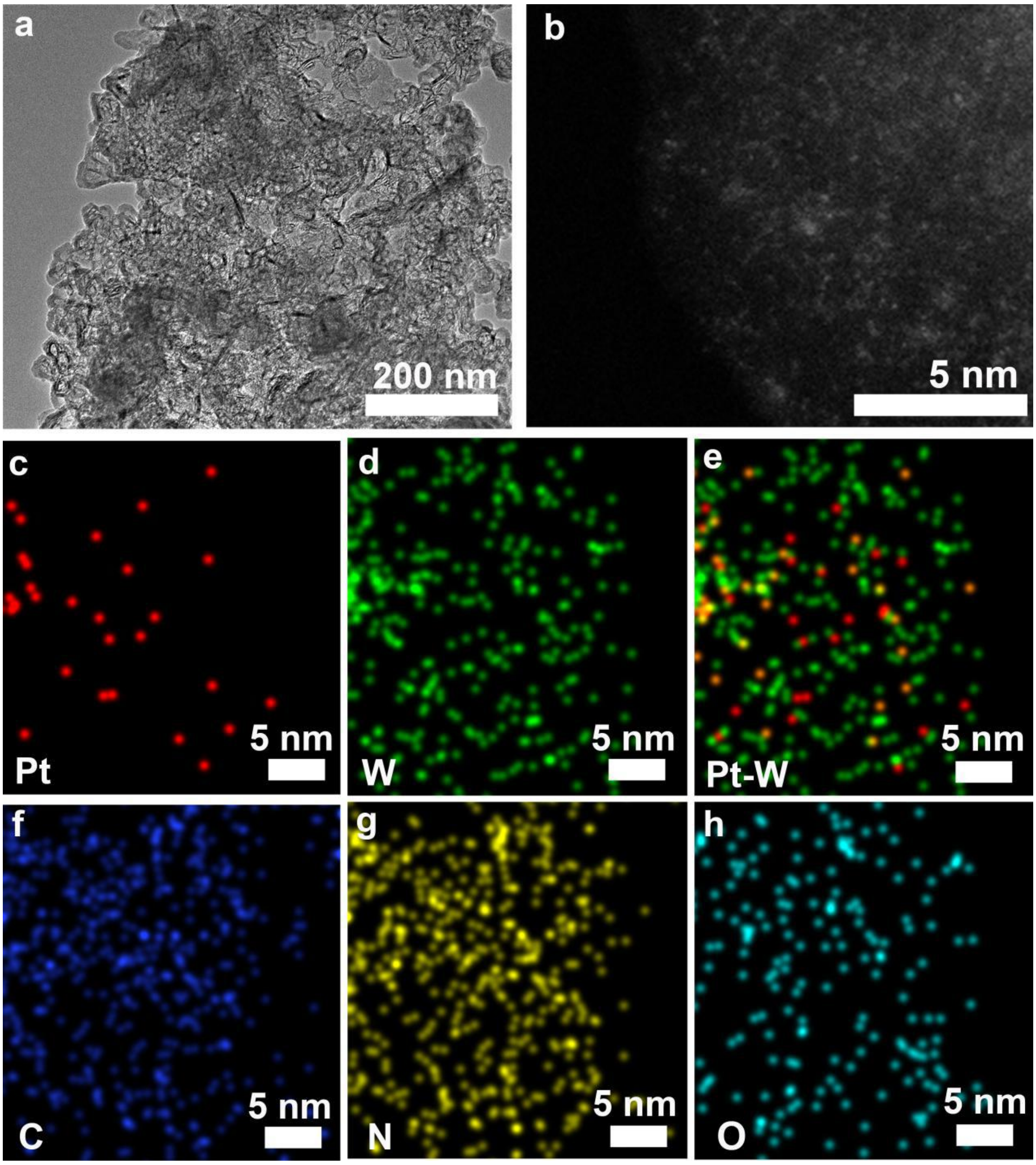

Supplementary Figure 39: TEM and HAAD-STEM of $Pt_2(W_5O_{18})_2/C$. (a) TEM of 1% $Pt_2(W_5O_{18})_2/C$. **(b)** STEM of 1% $Pt_2(W_5O_{18})_2/C$. **(c-g)** Element mapping of 1% $Pt_2(W_5O_{18})_2/C$ of, Pt **(c)**, W **(d)**, Pt-W **(e),** C **(f),** N **(g)** and C **(g)**.

Supplementary Figure 39 shows homogeneous dispersion of crystal $Pt_2(W_5O_{18})_2/C$ on Ketjen black, and no Pt NPs or cluster were observed. The corresponding HAADF image displays isolated bright dots, which can be attributed to the heavy Pt and W atoms, further indicating $Pt_2(W_5O_{18})_2/C$ molecules were monodisperse. The elemental mapping verifies the presence of Pt, W, O and C elements. The above results demonstrate crystal $Pt_2(W_5O_{18})_2/C$ molecules are successfully monodispersed on Ketjen black.

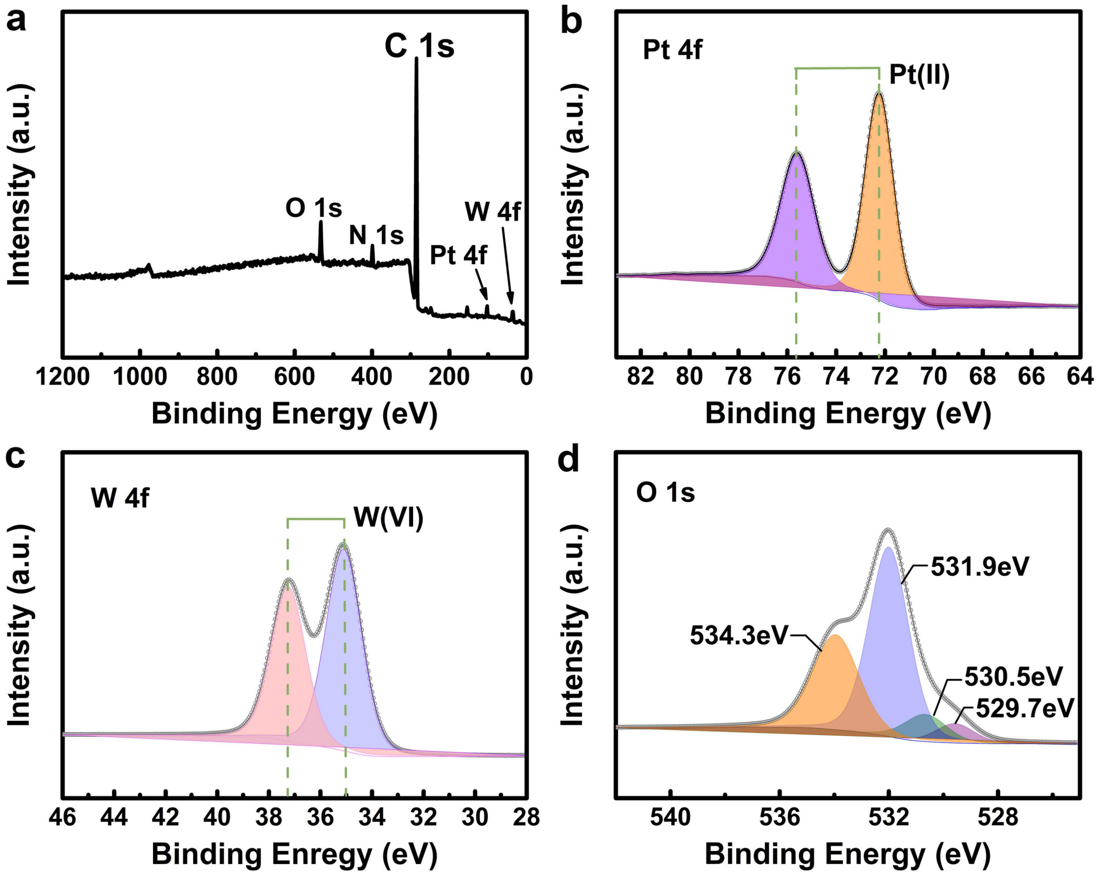

Supplementary Figure 40: XPS for $Pt_2(W_5O_{18})_2$/C. (a) The full XPS spectra for 1% $Pt_2(W_5O_{18})_2$/C. **(b)** High-resolution XPS spectra of Pt for 1% $Pt_2(W_5O_{18})_2$/C. **(c)** High-resolution XPS spectra of W for 1% $Pt_2(W_5O_{18})_2$/C. **(d)** High-resolution XPS spectra of O for 1% $Pt_2(W_5O_{18})_2$/C.

The full XPS spectrum of $Pt_2(W_5O_{18})_2$/C verifies the presence of Pt, W, O and C elements. Supplementary Figure 40 indicates the Pt $4f_{7/2}$ and Pt $4f_{5/2}$ located at 72.3 and 75.8 eV, respectively, which are consistent with the values of Pt(II) as reported in the literatures. It is notably that there is not existence of peak at 71.4 and 74.7 eV, implying the absence of metallic Pt. The W XPS spectra with two peaks at 34.9 and 37.1 eV assigned to $4f_{7/2}$ and $4f_{5/2}$ of W(VI). The O 1s spectra demonstrates the existence of W=O and W-O-W bond and crystal water absorbed on $Pt_2(W_5O_{18})_2$/C molecule with banding energies of 529.7 eV, 530.5 eV and 534.3 eV, respectively. The peak at 531.9 eV is assigned to oxygen present on the surface of carbon, which may come from oxygen absorbed on Ketjen black, and covers the peak of Pt-O bond.

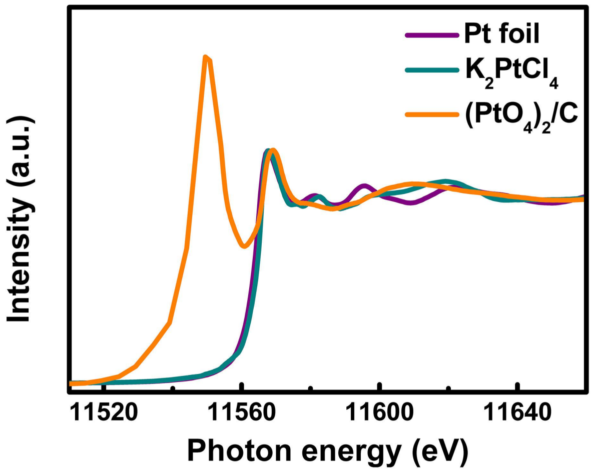

Supplementary Figure 41: Pt L_3-edge XANES spectra of $Pt_2(W_5O_{18})_2$/C. Pt L_3-edge XANES spectra of 1% $Pt_2(W_5O_{18})_2$/C, K_2PtCl_4 and Pt foil.

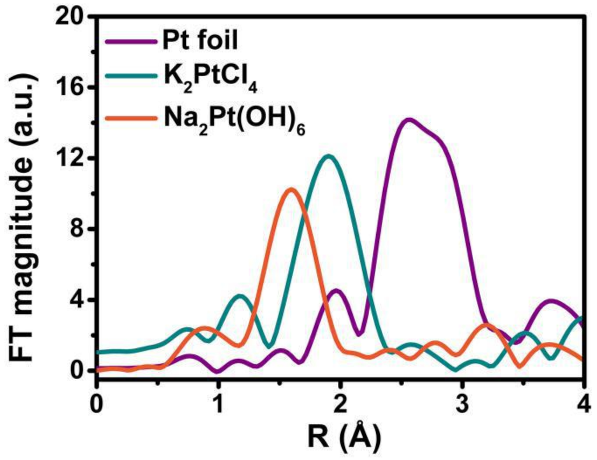

Supplementary Figure 42: The Fourier transform of the EXAFS data. The Fourier transform of the EXAFS data of $Na_2Pt(OH)_6$, K_2PtCl_4 and Pt foil.

The normalized XANES spectra of 1% $Pt_2(W_5O_{18})_2/C$ and reference materials (K_2PtCl_4 and Pt foil) was shown in Supplementary Figure 41. Although the peak intensity of K_2PtCl_4 is similar to that of Pt foil, the Fourier transformations (FT) of k^3-weighted for EXAFS oscillations for K_2PtCl_4 is quite different with those of Pt foil, which is correspond to the existence of Pt(II), because of the influence of their coordination environment. The Pt white-line intensity for 1% $Pt_2(W_5O_{18})_2/C$ is similar with K_2PtCl_4, which means the valence state of Pt remained unchanged during the preparation of catalyst, which is consistent with the results of XPS. Feature B is due to the interference of W L_2-edge, which is not affect the analysis of main peak of 1% $Pt_2(W_5O_{18})_2/C$.

Supplementary note 2

We further explore the HER performance of electrocatalyst $Pt_2(W_5O_{18})_2/C$. As shown in Supplementary Figure. 43a, the $[Pt_2(W_5O_{18})_2]^{8-}$ anion is composed of two Pt(II) ions capped by two lacunary Lindquist structures $(W_5O_{18}{}^{6})^{-}$. Each Pt(II) coordinates with the terminal oxygen atoms of W_5O_{18} fragment in a square plane environment. The distance of Pt-O bond varies within the scope of 1.984(6)-2.000(6) Å. The distance between two Pt atoms is 3.1315(8) Å, which is obviously longer than the metal-metal bond distance. The 1% $Pt_2(W_5O_{18})_2/C$ also exhibits an excellent electrocatalytic hydrogen evolution performance with an overpotential of 26 mV at 10 mA cm^{-2} (Supplementary Figure 43b and Figure 44), which is similar to that of 1% PtW_6O_{24}/C. The Tafel slope is 29.8 mV dec^{-1} with the Volmer-Tafel mechanism (Supplementary Figure 43c and Figure 45). The exchange current density is 1.42 mA cm^{-2} and the mass activity is 10.976 mg^{-1} at 77 mV, respectively. The TOFs at 100 mV is 16.63 s^{-1}. All these results are similar to 1% PtW_6O_{24}/C (Supplementary Figure 43b and Figures 46-49). Electrochemical impedance spectroscopy (EIS) (Supplementary Figure 50) suggests the charge transfer resistance of 1% $Pt_2(W_5O_{18})_2/C$, implying a fast HER kinetics. In addition, 1% $Pt_2(W_5O_{18})_2/C$ also possesses an excellent FE (nearly 100%) (Supplementary Figure 51), good anti-toxicity (Supplementary Figure

52) and stability during the whole HER process (Supplementary Figure 43d, Figures. 53-56 and Tables 4-5). These observations further confirm that Pt-O bond can be a more active site than metallic Pt^0 toward electrocatalytic HER.

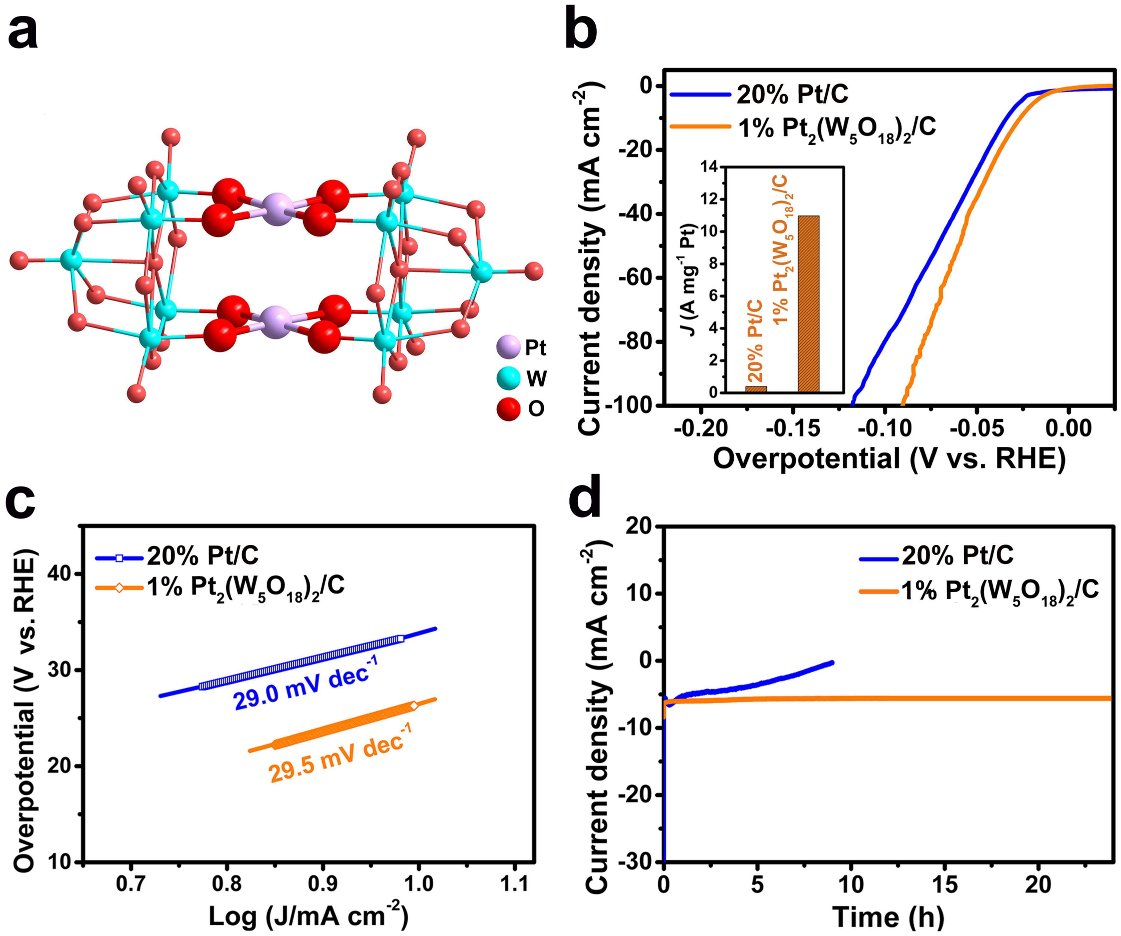

Supplementary Figure 43: Structure and HER performance of the $Pt_2(W_5O_{18})_2/C$ catalyst. (a) The ball and stick representation of **$Pt_2(W_5O_{18})_2$**. **(b)** The polarization curves of 1% $Pt_2(W_5O_{18})_2/C$ and 20% Pt/C at a current density of 10 mA cm^{-2} in N_2-saturated 0.5 M H_2SO_4. Inset: mass activity of 1% $Pt_2(W_5O_{18})_2/C$ and 20% Pt/C at 77 mV. **(c)** Tafel slope of 1% $Pt_2(W_5O_{18})_2/C$ and 20% Pt/C. **(d)** Time-dependent current density current of 1% $Pt_2(W_5O_{18})_2/C$ and 20% Pt/C within 24 h.

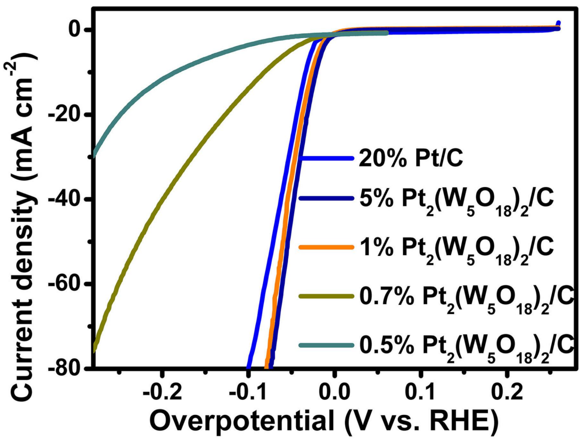

Supplementary Figure 44: LSV of $Pt_2(W_5O_{18})_2/C$. The HER polarization curves of $Pt_2(W_5O_{18})_2/C$ in 0.5 M H_2SO_4 at rate of 5 mV s^{-1}.

Series of $Pt_2(W_5O_{18})_2/C$ with different loading of Pt have been prepared. With the increase of Pt loading, the HER performance of catalysts was obviously enhanced. When the loading of Pt increase to 5%, $Pt_2(W_5O_{18})_2$ are aggregated, resulting in a decrease of catalyst utilization and a slight enhancement in HER performance which is compared with 1% $Pt_2(W_5O_{18})_2/C$.

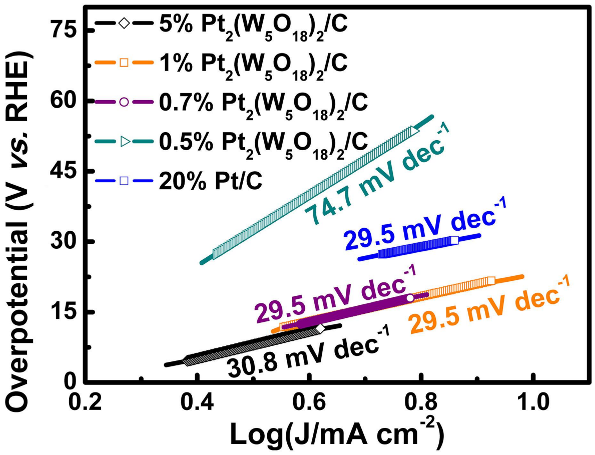

Supplementary Figure 45: Tafel slope of $Pt_2(W_5O_{18})_2$/C. Tafel slope of $Pt_2(W_5O_{18})_2$/C with different amounts of $Pt_2(W_5O_{18})_2$/C under the optimal conditions.

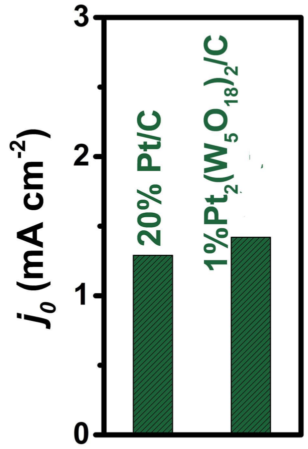

Supplementary Figure 46: The exchange current density of $Pt_2(W_5O_{18})_2/C$. The exchange current density of 1% $Pt_2(W_5O_{18})_2/C$ and 20% Pt/C. The exchange current density of 1% $Pt_2(W_5O_{18})_2/C$ is 1.42 mA cm^{-2}, which is similar to 1% PtW_6O_{24}/C, is far high that of 20% Pt/C.

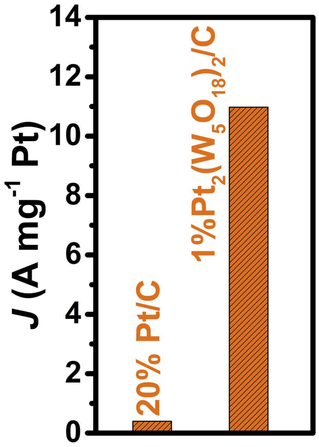

Supplementary Figure 47: The mass activity of $Pt_2(W_5O_{18})_2$/C. The mass activity of 1% $Pt_2(W_5O_{18})_2$/C and 20% Pt/C at 77 mV. The mass activity of 1% $Pt_2(W_5O_{18})_2$/C is 10.976 A mg^{-1}.

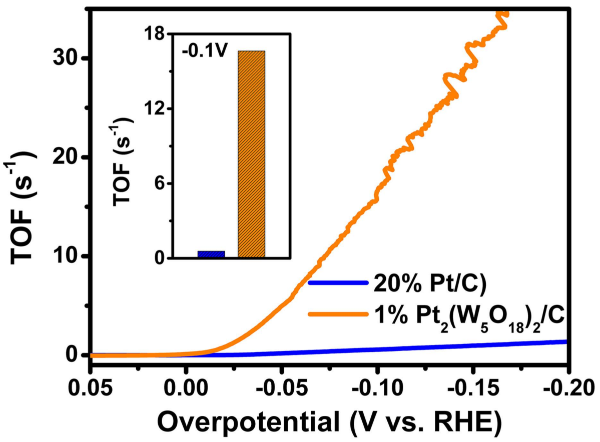

Supplementary Figure 48: TOF of 1% $Pt_2(W_5O_{18})_2/C$ and 20% Pt/C. The mass activity of 1% $Pt_2(W_5O_{18})_2/C$ is 16.63 s^{-1}.

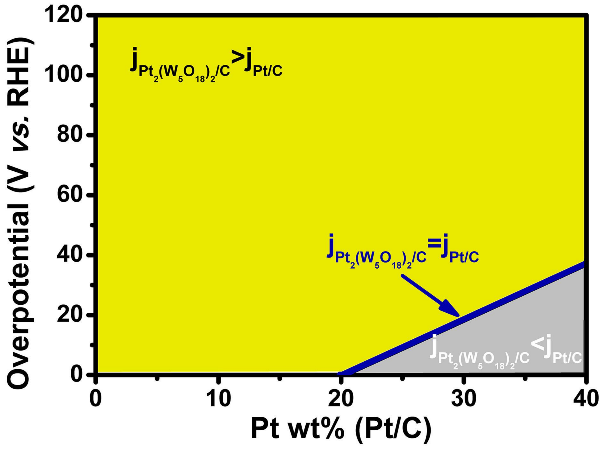

Supplementary Figure 49: The relationship between the overpotential, the current density and the Pt content. The relationship between the overpotential, the current density and the Pt content in Pt/C of 1% $Pt_2(W_5O_{18})_2/C$ and Pt/C.

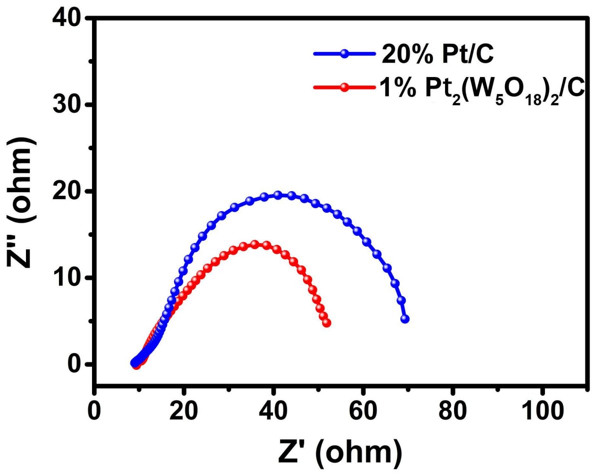

Supplementary Figure 50: The Nyquist plot of $Pt_2(W_5O_{18})_2/C$. The Nyquist plot of 1% $Pt_2(W_5O_{18})_2/C$ and 20% Pt/C at the overpotential of 40 mV vs RHE, suggesting the charge transfer resistance of 1% $Pt_2(W_5O_{18})_2/C$ is lower than that of 20% Pt/C, implying a fast HER kinetics for 1% $Pt_2(W_5O_{18})_2/C$, which is similar to 1% PtW_6O_{24}/C.

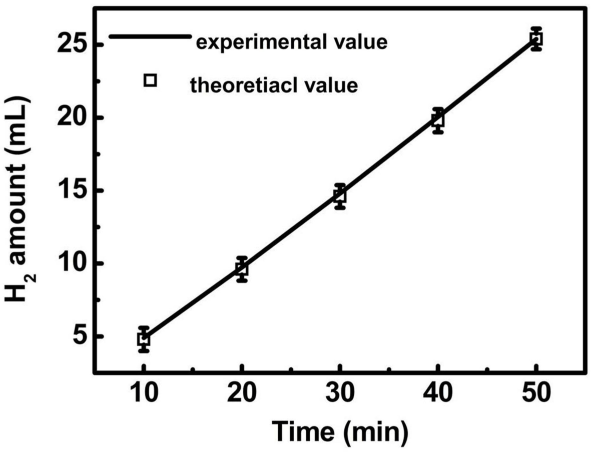

Supplementary Figure 51: The FE of $Pt_2(W_5O_{18})_2$/C. The Faradic efficient of 1% $Pt_2(W_5O_{18})_2$/C.

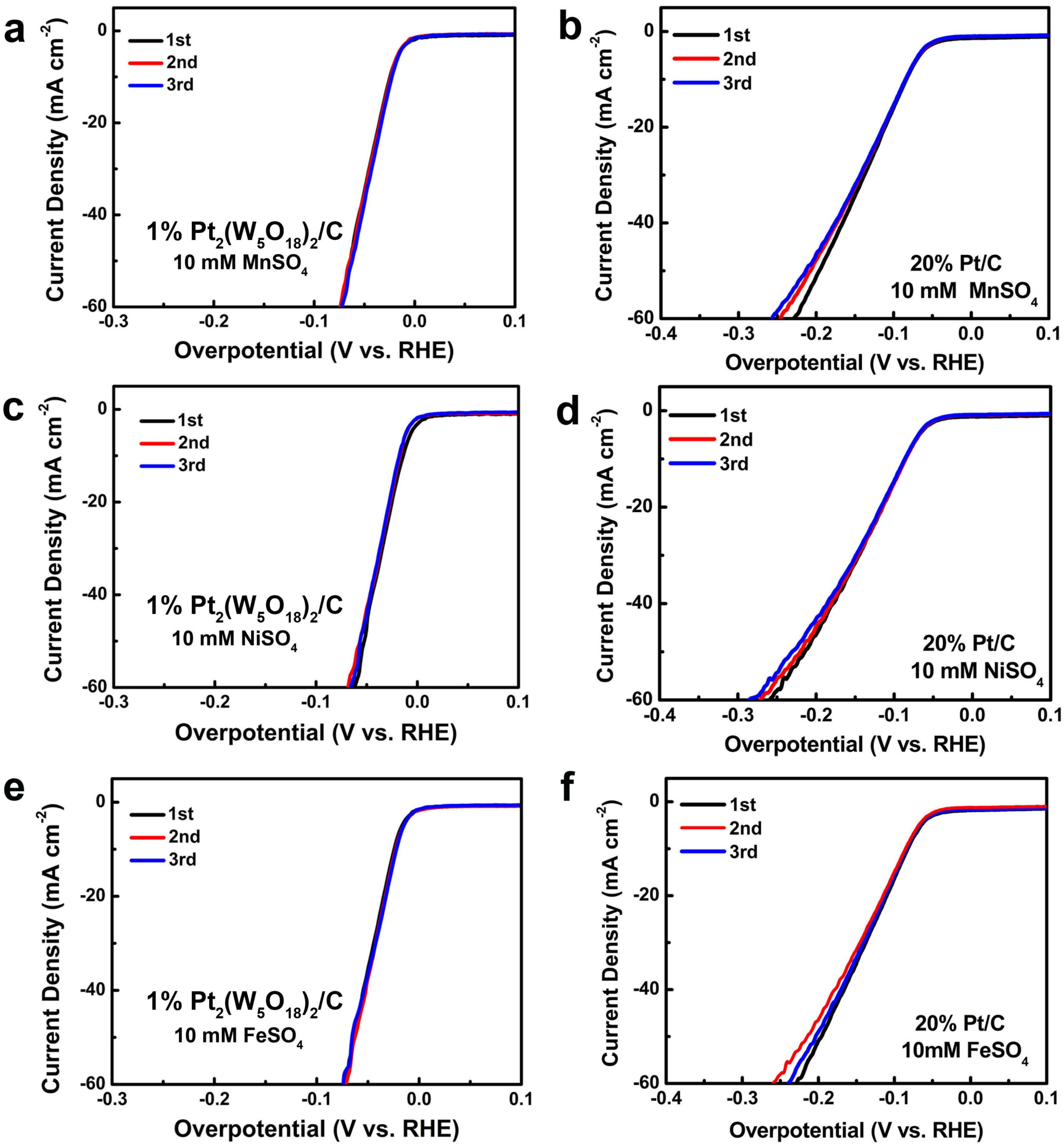

Supplementary Figure 52: Anti-toxicity test of $Pt_2(W_5O_{18})_2$/C and 20% Pt/C. (a) and **(b)** The HER polarization curves of 1% $Pt_2(W_5O_{18})_2$/C and 20% Pt/C in 10 mM $MnSO_4$. **(c)** and **(d)** The HER polarization curves of 1% $Pt_2(W_5O_{18})_2$/C and 20% Pt/C in 10 mM $NiSO_4$. **(e)** and **(f)** The HER polarization curves of 1% $Pt_2(W_5O_{18})_2$/C and 20% Pt/C in 10 mM $FeSO_4$.

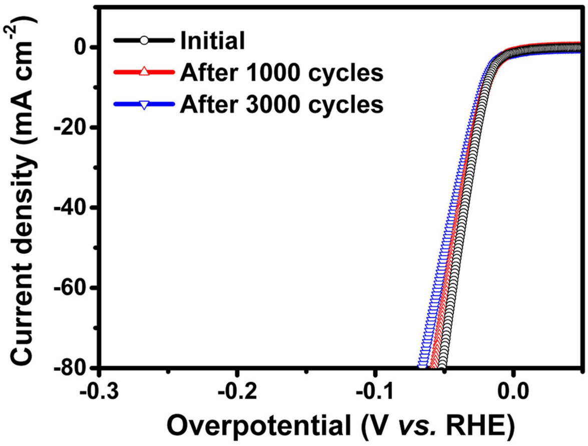

Supplementary Figure 53: LSV of $Pt_2(W_5O_{18})_2$/C before and after CV. The polarization curves of 1% $Pt_2(W_5O_{18})_2$/C before and after 1000 and 3000 cycles in 0.5 M H_2SO_4 at scan rate of 5 mV s^{-1}.

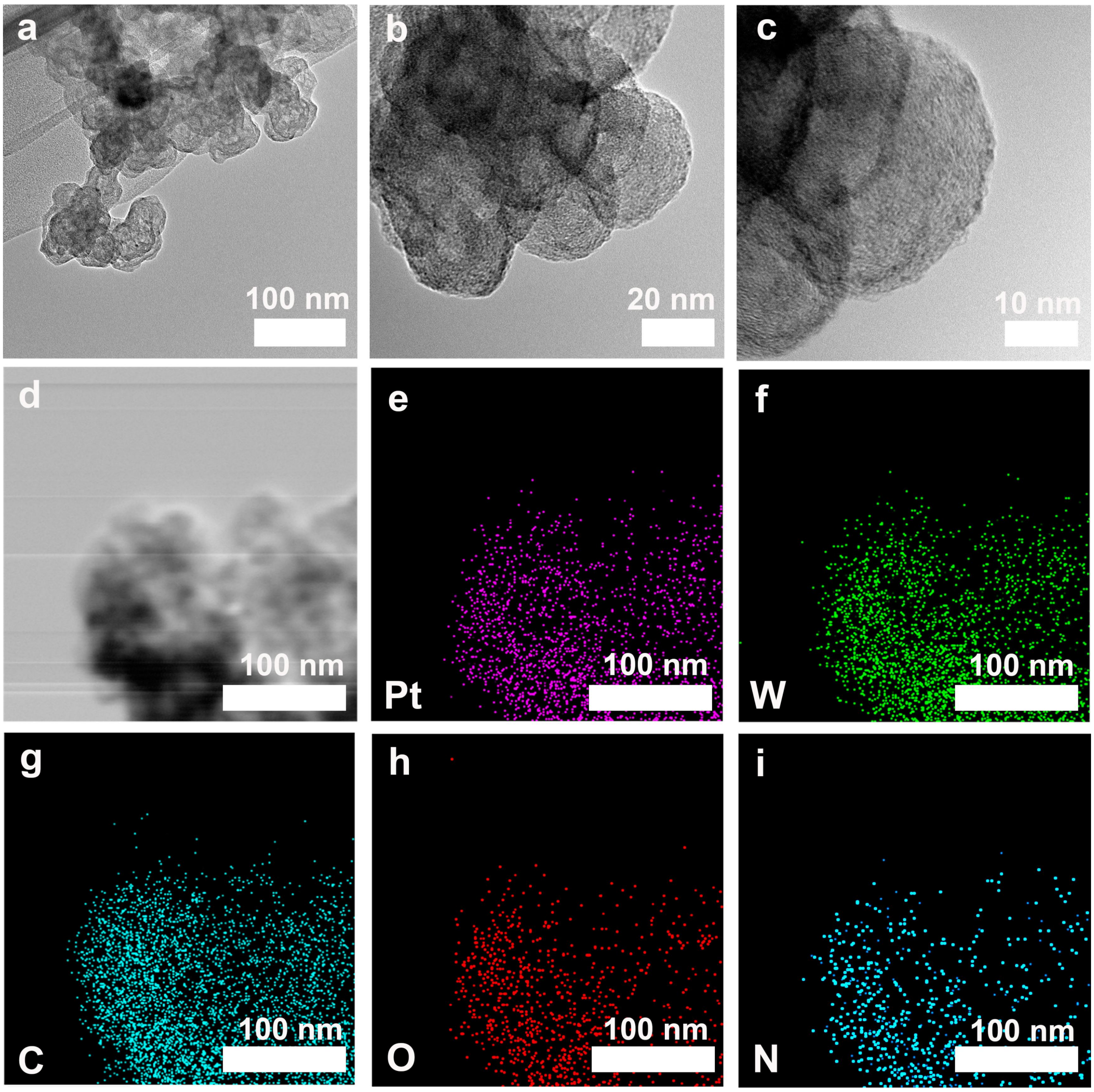

Supplementary Figure 54: TEM of $Pt_2(W_5O_{18})_2/C$ before and after HER. (a) TEM images of 1% $Pt_2(W_5O_{18})_2/C$ after electrochemical tests. **(b-c)** HRTEM images of 1% $Pt_2(W_5O_{18})_2/C$ after electrochemical tests. **(d-i)** Elemental mapping of 1% $Pt_2(W_5O_{18})_2/C$ after electrochemical tests. The TEM images of 1% $Pt_2(W_5O_{18})_2/C$ after long-term electrochemical test demonstrate the good stability of 1% $Pt_2(W_5O_{18})_2/C$, demonstrating the its morphology stays the same.

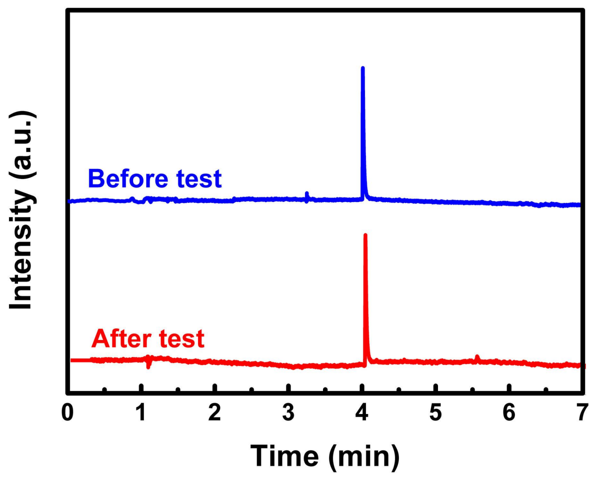

Supplementary Figure 55: The CE of $Pt_2(W_5O_{18})_2$ before and after HER. An electropherogram for 0.25 mM of $Pt_2(W_5O_{18})_2$ in a 20 mM NaH_2PO-H_3PO_4 solution (pH=3) before electrochemical reaction (blue line) and after electrochemical reaction (red line).

The capillary electrophoretic method was used to study the electrochemical stability of $Pt_2(W_5O_{18})_2$. This technique was investigated to separate species according to their charge-size ratios in small electrolyte-filled capillaries. As shown in the Supplementary Figure 55, the peak positions of $Pt_2(W_5O_{18})_2$ have no change before and after the electrochemical reaction, meaning the crystal structure is stable during the HER in acidic solution

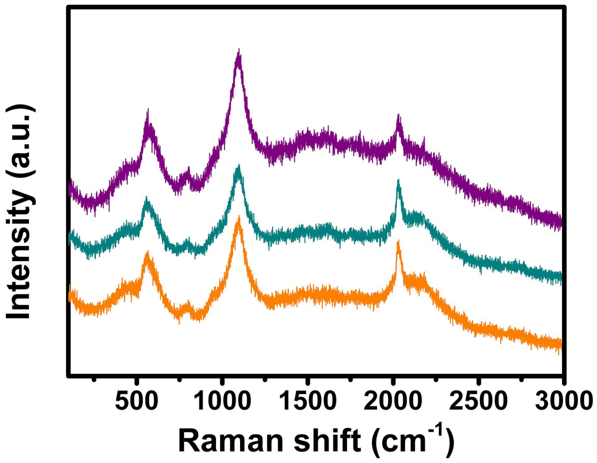

Supplementary Figure 56: Raman of $Pt_2(W_5O_{18})_2$. Raman of pure crystal $Pt_2(W_5O_{18})_2$ (origin), 1% $Pt_2(W_5O_{18})_2$/C before electrochemical hydrogen evolution reaction (dark cyan) and 1% $Pt_2(W_5O_{18})_2$/C after electrochemical hydrogen evolution reaction (purple).

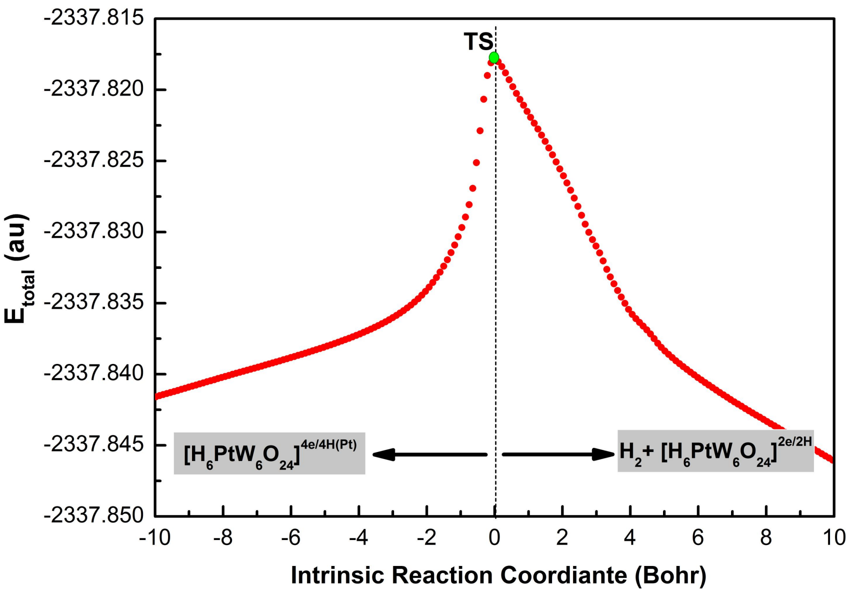

Supplementary Figure 57 IRC calculation for the transition state involved in Fig.3b. Intrinsic reaction coordinate (IRC) between $[H_6PtW_6O_{24}]^{4e/4H(Pt)}$ and H_2+$[H_6PtW_6O_{24}]^{2e/2H}$ through the transition states (TS) which were obtained by using UM06/6-31G(d,p) level of theory.

Supplementary Table 1 Crystal data of PtW_6O_{24} with different pH during synthesis[a].

	$Na_3[H_5PtW_6O_{24}] \cdot 17H_2O$	$Na_5[H_3PtW_6O_{24}] \cdot 20H_2O$
pH (during synthesis)	2.12	6.2
Formula weight	2062.47	2160.14
Crystal system	Triclinic	Triclinic
space group	*P*-1	*P*-1
a [Å]	10.7545(6)	10.576
b[Å]	12.4600(7)	12.551
c[Å]	16.2068(9)	10.027
α [°]	71.565(2)	115.06
β [°]	78.856(2)	99.51
γ [°]	70.940(2)	62.32
V [Å3]	1937.50(19)	1066.9
Z	2	1
$D_{calcd.}$ [gcm^{-3}]	3.535	3.362
μ [mm–1]	21.482	20.65
Temperature [K]	296(2)	293

a. Lee, U. *et al.* Structure of Pentasodium Trihydrogenhexatungstoplatinate(IV) Icosahydrate, $Na_5[H_3PtW_6O_{24}]\cdot 20H_2O$. *Acta. Cryst.* **39**, 817-819 (1983)

Supplementary Table 2 The ICP result of Pt content in PtW_6O_{24}/C before HER test.

Samples	Platinum content (μg/mL)	Percentage by weight %
**5% PtW_6O_{24}/C**	5.12	5.68
**1% PtW_6O_{24}/C**	0.95	0.10
**0.7% PtW_6O_{24}/C**	0.63	0. 7
**0.5% PtW_6O_{24}/C**	0.52	0.57

Supplementary Table 3 The ICP result of Pt content in PtW_6O_{24}/C after HER test

Samples	Platinum content (μg/mL)	Percentage by weight %
**5% PtW_6O_{24}/C**	5.02	5.57
**1% PtW_6O_{24}/C**	0.93	0.10
**0.7% PtW_6O_{24}/C**	0.62	0. 69
**0.5% PtW_6O_{24}/C**	0.50	0.55

Supplementary Table 4 The ICP result of Pt content in $Pt_2(W_5O_{18})_2$ /C before HER test.

**Samples**	**Platinum content (μg/mL)**	**Percentage by weight %**
**5% $Pt_2(W_5O_{18})_2$ /C**	5.26	4.8
**1% $Pt_2(W_5O_{18})_2$**	1.09	0.99
**0.7% $Pt_2(W_5O_{18})_2$ /C**	0.69	0. 67
**0.5% $Pt_2(W_5O_{18})_2$ /C**	0.51	0.46

Supplementary Table 5 The ICP result of Pt content in $Pt_2(W_5O_{18})_2$ /C after HER test

**Samples**	**Platinum content (μg/mL)**	**Percentage by weight %**
**5% $Pt_2(W_5O_{18})_2$ /C**	5.19	4.7
**1% $Pt_2(W_5O_{18})_2$ /C**	0.98	0.91
**0.7% $Pt_2(W_5O_{18})_2$ /C**	0.66	0. 6
**0.5% $Pt_2(W_5O_{18})_2$ /C**	0.49	0.45

Supplementary Table 6 Calculated Gibbs free energy (eV) for stepwise protonation of $[PtW_6O_{24}]^{8-}$ and associated reduction potential (V).

Protonation and reduction step	Δ*G* (eV)	*E* (V)
$[PtW_6O_{24}]^{8-}$ + H^+ → $[HPtW_6O_{24}]^{7-}$	−1.97	--
$[HPtW_6O_{24}]^{7-}$ + H^+ → $[H_2PtW_6O_{24}]^{6-}$	−1.75	--
$[H_2PtW_6O_{24}]^{6-}$ + H^+ → $[H_3PtW_6O_{24}]^{5-}$	−1.38	--
$[H_3PtW_6O_{24}]^{5-}$ + H^+ → $[H_4PtW_6O_{24}]^{4-}$	−0.86	--
$[H_4PtW_6O_{24}]^{4-}$ + H^+ → $[H_5PtW_6O_{24}]^{3-}$	−0.46	--
$[H_5PtW_6O_{24}]^{3-}$ + H^+ → $[H_6PtW_6O_{24}]^{2-}$	−0.03	--
$[H_6PtW_6O_{24}]^{2-}$ + H^+ → $[H_7PtW_6O_{24}]^{1-}$	0.41	--
$[H_6PtW_6O_{24}]^{2-}$ + e^- → $[H_6PtW_6O_{24}]^{3-}$		1.12
$[H_6PtW_6O_{24}]^{3-}$ + H^+ → $[H_7PtW_6O_{24}]^{2-}$	−0.13	--
$[H_7PtW_6O_{24}]^{2-}$ + e^- → $[H_7PtW_6O_{24}]^{3-}$		0.82
$[H_7PtW_6O_{24}]^{3-}$ + H^+ → $[H_8PtW_6O_{24}]^{2-}$	−0.45	--
$[H_8PtW_6O_{24}]^{2-}$ + e^- → $[H_8PtW_6O_{24}]^{3-}$		−1.47
$[H_8PtW_6O_{24}]^{3-}$ + H^+ → $[H_9PtW_6O_{24}]^{2-}$	−0.67	--
$[H_9PtW_6O_{24}]^{2-}$ + e^- → $[H_9PtW_6O_{24}]^{3-}$		−1.20
$[H_9PtW_6O_{24}]^{3-}$ + H^+ → $[H_{10}PtW_6O_{24}]^{2-}$	−0.33	

a) The absolute aqueous Gibbs free energy of the proton (H^+) was selected from the data determined by Coe and co-workers,[6] which is -11.46 eV. For species with different protonated isomers, only the most stable one was given, and all the six protons are identified to adsorb on the triply-bridging oxygen (μ_3-O).

b) The reduction potential (*E*) was theoretically determined by computing the free energy associated with the process $POM_{ox}(aq) + ne- \rightarrow POM_{red}(aq)$ and using the relation $E_{NHE} = -\Delta G_{ox|red}/nF - E_{SHE}$), where $\Delta G_{ox|red}$ is the free energy change of the reduction process, F is the Faraday constant, and E_{SHE} corresponds to the standard hydrogen electrode ($E_{SHE} = 4.24V$), pH effect is neglected here.

Since the Anderson POMs (M =W, Mo) with Pt^{IV}-centered generally exhibits a mixed A-B type structure (number of protons between 2 to 5.5) depending on pH conditions. The proton distribution of $[PtW_6O_{24}]$ was analyzed in Table S6 by DFT calculations. The Gibbs free energy change for stepwise protonation of unprotonated $[PtW_6O_{24}]^{8-}$ to sixth-protonated state are all exothermic. Even the sixth-protonation step is an exothermic process with energy of only -0.03 eV, this may still allow it to occur under low pH condition. This was confirmed by experiment, which shows the $[PtW_6O_{24}]$ was fifth-protonated in aqueous solution with pH = 3.5. We thus proposed the six protonated state $[H_6PtW_6O_{24}]$ could be the initially dominant species in 0.5 M H_2SO_4 (pH = 0) before electrocatalytic process. Starting from $[H_6PtW_6O_{24}]^{2-}$, the direct protonation is limited from thermodynamic view, and it may proceed by a reduction step or proton-electron coupled process to obtain $[H_6PtW_6O_{24}]^{1e/1H}$.

Supplementary Table 7 Comparison of HER performance of different reported materials in acid media.

Catalyst	Working Electrode	Overpotential (10 mA cm^{-2})	Tafel slope	Reference
**PtW_6O_{24}/C**	glassy carbon electrode	22	29.8	This work
**$Pt_2(W_5O_{18})_2$ /C**	glassy carbon electrode	26	29.5	This work
**Commercial Pt/C**	glassy carbon electrode	33	29	This work
**$Mo_2TiC_2T_x$-Pt_{SA}**	Carbon paper	30	73	1
**Pt@PCM**	glassy carbon electrode	106	65.3	2
**ALD50Pt/NGNs**	glassy carbon electrode	39	29	3
**W-SAC**	glassy carbon electrode	105	58	4
**HUST-100**	glassy carbon electrode	234	82	5
**WO_x@C/C-2-900**	glassy carbon electrode	36	19.17	6
**er-WS_2-Pt**	glassy carbon electrode	40	27	7
**400-SWNT/Pt**	glassy carbon electrode	27	38	8
**Pt-MoS_2**	glassy carbon electrode	53	40	9
**Rh/SiNW**	glassy carbon electrode	85	24	10
**PtML/Au NF/Ni foam**	glassy carbon electrode	60	53	11
**Pt-GT-1**	glassy carbon electrode	18	24	12
**Pt-MoS_2 (0.1 M H_2SO_4)**	glassy carbon electrode	145	96	13
**$Mo_1N_1C_2$**	glassy carbon electrode	154	86	14
**Co_1/PCN**	glassy carbon electrode	151	52	15
**PtCoFe@CN**	glassy carbon electrode	40	29	16

Supplementary Table 8 Optimized coordinates (xyz) for all related species

$[PtW_6O_{24}]$

-8 1 (represent charge and multiplicity spin)

W	2.86550500	0.71146800	1.42719900
W	-2.59530200	1.93239400	0.43522800
W	0.25826100	2.64075400	1.89805000
O	3.24924900	0.73816800	3.14304300
O	4.38764800	1.15494500	0.66658400
O	-2.82771200	3.60233300	-0.06530200
O	-4.10502300	1.54564800	1.24655800
O	0.14156900	4.34004800	1.45859500
O	0.56324200	2.69492500	3.62766300
O	-2.97223400	1.23747000	-1.36136500
O	2.10142000	2.50384500	1.22568200
O	-1.63953000	2.20375300	2.13266300
O	-1.98078100	-0.14645700	0.43165000
O	-0.47837600	1.98104500	-0.03509000
O	0.73689000	0.52702000	1.81335300
W	-2.85343400	-0.71956900	-1.47256300
W	2.60712900	-1.94100600	-0.47984100
W	-0.24632100	-2.64918000	-1.94301200
O	-3.23725400	-0.74679200	-3.18835600
O	-4.37542900	-1.16328300	-0.71174800
O	2.83964500	-3.61104800	0.02022300
O	4.11664800	-1.55408800	-1.29152100
O	-0.12954500	-4.34843900	-1.50347000
O	-0.55148200	-2.70347600	-3.67258500
O	2.98418600	-1.24591900	1.31633000
O	-2.08925000	-2.51201600	-1.27041800
O	1.65128900	-2.21202700	-2.17753600
O	1.99282600	0.13843900	-0.47643900
O	0.49050200	-1.98924800	-0.00974700
O	-0.72501500	-0.53518100	-1.85837900
Pt	0.00599600	-0.00408600	-0.02248300

$[H_1PtW_6O_{24}]$

-7 1

W	2.87064100	0.71262200	1.43781100
W	-2.58574900	2.05046200	0.43534800
W	0.26631500	2.65982400	1.89956700
O	3.28170900	0.76415500	3.13879800
O	4.36166100	1.16117800	0.63269700
O	-2.85379600	3.69345600	-0.07600000
O	-4.10065800	1.58467900	1.17938000
O	0.14964100	4.35444800	1.47436100
O	0.54247000	2.67569600	3.62896000
O	-2.81922700	1.17121000	-1.28916000
O	2.08467200	2.50208800	1.22833600
O	-1.67925200	2.28151000	2.12914700
O	-1.97790500	-0.25304700	0.58212300
O	-0.49927800	1.94526300	0.01903700
O	0.75119500	0.49652400	1.83567700
W	-2.84879800	-0.75735300	-1.57258800
W	2.61283200	-1.95205700	-0.48742600
W	-0.23885100	-2.66338400	-1.95786300
O	-3.26852800	-0.76726100	-3.26193900
O	-4.35539300	-1.12553800	-0.75651300
O	2.86823600	-3.61988100	-0.01775400
O	4.09686400	-1.51782400	-1.30995500
O	-0.12875600	-4.34783200	-1.48531100
O	-0.55899200	-2.72706900	-3.67695100
O	2.99007000	-1.23115600	1.29036600
O	-2.12123600	-2.52820200	-1.31387300
O	1.63697300	-2.21087500	-2.17966100
O	1.93630700	0.15833600	-0.50324000
O	0.50841800	-2.00396200	0.02118800
O	-0.73941500	-0.57212900	-1.80818100
Pt	0.02921600	-0.04169400	0.02491200
H	-2.07964300	-0.80161400	1.36615900

$[H_2PtW_6O_{24}]$
-6 1

W	2.87723600	0.70051200	1.46051400
W	-2.59279800	2.04987100	0.43057900
W	0.27160000	2.67941500	1.90143300
O	3.27597500	0.68723400	3.16033700
O	4.36597500	1.14337400	0.66504600
O	-2.77514400	3.68602300	-0.11930200
O	-4.12290500	1.67522800	1.18286300
O	0.13671900	4.33096500	1.35794200
O	0.53699800	2.81096400	3.62201600
O	-2.84790700	1.13656700	-1.27372800
O	2.09644800	2.47588200	1.25507000
O	-1.65691400	2.27410000	2.10400700
O	-2.00748900	-0.23610300	0.64698500
O	-0.46975300	1.88590500	-0.02456200
O	0.71612500	0.50281500	1.88432100
W	-2.88188700	-0.76963700	-1.60025200
W	2.70056400	-1.90007000	-0.52037000
W	-0.29906800	-2.66383500	-2.08497900
O	-3.31517600	-0.72384300	-3.27834800
O	-4.35531000	-1.20370700	-0.77056800
O	2.87838600	-3.58766800	-0.11169700
O	4.17524500	-1.48692100	-1.33811300
O	-0.09203400	-4.31878300	-1.57300200
O	-0.53267300	-2.75768900	-3.80144200
O	3.02902000	-1.26304200	1.27265600
O	-2.15129000	-2.58300300	-1.45900700
O	1.54407900	-2.08314500	-2.07717100
O	1.90118300	0.11668400	-0.43355800
O	0.45325700	-2.07262600	0.18877200
O	-0.77263500	-0.62404800	-1.73668900
Pt	-0.01204500	-0.04990300	0.10234500
H	-2.10026500	-0.66864000	1.50310700
H	0.26190200	-2.45556500	1.05203100

$[H_3PtW_6O_{24}]$
-5 1

W	2.88551200	0.74607500	1.57714000
W	-2.72704700	1.90940200	0.49480300
W	0.29218200	2.66208300	2.04786300
O	3.22938100	0.70508200	3.27187900
O	4.39181400	1.16416900	0.81521200
O	-2.91085900	3.57638900	0.03423800
O	-4.18162200	1.50202600	1.33779600
O	0.10944100	4.33765900	1.61766600
O	0.54175300	2.65499600	3.75937500
O	-3.13373100	1.21376700	-1.28314600
O	2.13129500	2.53877300	1.41028200
O	-1.55797700	2.09912700	2.02512000
O	-1.86291600	-0.07349300	0.34566800
O	-0.41783000	2.12537100	-0.21611300
O	0.72323000	0.56980600	1.65696500
W	-2.98078200	-0.72663500	-1.39986000
W	2.61473400	-2.07566600	-0.44255700
W	-0.22521500	-2.79720700	-1.86496600
O	-3.33087300	-0.78898000	-3.10248900
O	-4.44627300	-1.20600500	-0.61512300
O	2.79201700	-3.70280800	0.11640200
O	4.12633000	-1.69034000	-1.21220500
O	-0.11999900	-4.43687300	-1.32494200
O	-0.57287900	-2.89973400	-3.56616500
O	2.84422100	-1.16199300	1.25064500
O	-1.98728800	-2.37600000	-1.18487800
O	1.66311700	-2.37368200	-2.12064100
O	2.05586000	0.27021800	-0.65667500
O	0.48984600	-1.83012600	-0.05326300
O	-0.66235000	-0.41483900	-2.03221300
Pt	0.05743200	0.11550700	-0.17027700
H	-0.35082900	0.15887800	-2.74238700
H	-0.12329900	2.57441000	-1.01745900
H	2.22332300	0.80713500	-1.44030400

$[H_4PtW_6O_{24}]$

-4 1

W	3.00019200	0.71851400	1.38792900
W	-2.62843800	2.07888900	0.43348700
W	0.22064800	2.79694000	1.86459600
O	3.31249100	0.74744600	3.09347400
O	4.46833000	1.22764400	0.63856700
O	-2.79219700	3.69811400	-0.13806300
O	-4.14273600	1.69065800	1.18078300
O	0.12429600	4.43967000	1.34847000
O	0.55573500	2.86048200	3.56523400
O	-2.88218800	1.15311300	-1.28246300
O	1.97399800	2.34473000	1.19817600
O	-1.67285500	2.36234900	2.09312800
O	-2.04820400	-0.25219700	0.59492300
O	-0.47316300	1.83779800	0.02682400
O	0.62553100	0.34812100	1.99571800
W	-2.96799200	-0.73322200	-1.55278700
W	2.75358700	-1.93628400	-0.52003000
W	-0.24546900	-2.75754400	-2.04230200
O	-3.23761600	-0.76000900	-3.25525800
O	-4.48234100	-1.17448300	-0.85606600
O	2.93134500	-3.59948700	-0.06933300
O	4.18682600	-1.53539900	-1.39356300
O	-0.11049100	-4.43537300	-1.66622000
O	-0.57932700	-2.70395500	-3.73231100
O	3.16129600	-1.22367700	1.23274100
O	-2.00203200	-2.39817800	-1.30647400
O	1.56296800	-2.15660000	-2.06636100
O	1.87949900	0.08355800	-0.37904900
O	0.44458900	-2.11791900	0.15473700
O	-0.62366900	-0.42461100	-1.78137500
Pt	-0.02541600	-0.08867200	0.14251100
H	-0.22510000	0.25684500	-2.34215000
H	-2.23363100	-0.80840400	1.36257100
H	0.28449900	-0.25552800	2.66930300
H	0.13340500	-2.58421000	0.94132800

[$H_5PtW_6O_{24}$]
-3 1

W	3.00530700	0.72779600	1.50229700
W	-2.63271600	2.09264200	0.41543200
W	0.23474800	2.81440100	1.87094600
O	3.32760800	0.73190600	3.19244100
O	4.49299400	1.15912600	0.75552200
O	-2.81690400	3.70273800	-0.16117200
O	-4.12412500	1.69118900	1.18955800
O	0.16648600	4.44952000	1.34160900
O	0.56917900	2.87532700	3.56500100
O	-2.88899700	1.11477100	-1.26202500
O	2.02173800	2.34200100	1.23047000
O	-1.64548400	2.37954800	2.06205600
O	-1.97240100	-0.26494400	0.61293300
O	-0.45906200	1.86648100	-0.02411300
O	0.64252700	0.37894200	1.94114400
W	-2.98530300	-0.77269900	-1.53561900
W	2.75202400	-2.05297700	-0.53348500
W	-0.24354700	-2.78450300	-2.06352100
O	-3.32341900	-0.81913900	-3.22044200
O	-4.45658300	-1.21579600	-0.76127400
O	2.99468200	-3.69252900	-0.08154300
O	4.19868500	-1.58558300	-1.33570900
O	-0.10343100	-4.46077400	-1.71169600
O	-0.56720200	-2.69444900	-3.74927200
O	2.95077200	-1.18796500	1.17858400
O	-1.97914000	-2.41976400	-1.30926300
O	1.59776700	-2.20050700	-2.07555000
O	1.88498200	0.22126000	-0.52797200
O	0.46415200	-2.09267800	0.09666000
O	-0.65988500	-0.40420000	-1.81417300
Pt	-0.03475700	-0.04909600	0.08641000
H	-0.40112100	0.33302700	-2.38858500
H	1.91581900	0.89159500	-1.22811800
H	-2.10517400	-0.81109800	1.40137600
H	0.32593200	-0.23530400	2.61969500
H	0.16807100	-2.55613200	0.89286600

$[H_6PtW_6O_{24}]$

-2 1

W	3.03522900	0.75180000	1.51256400
W	-2.75194900	2.05192500	0.46346100
W	0.27556800	2.80090200	2.00934400
O	3.37349400	0.75841400	3.19386400
O	4.50036400	1.19712700	0.74034500
O	-2.93196900	3.68185700	-0.03812200
O	-4.21060200	1.64789200	1.26964800
O	0.14183300	4.44601300	1.54471100
O	0.56193600	2.81524700	3.70009900
O	-2.95309200	1.14462600	-1.23215300
O	2.02114300	2.38538100	1.29368300
O	-1.56359500	2.21291800	1.97797900
O	-1.93088900	-0.22757900	0.53271800
O	-0.40980000	1.97547700	-0.17540800
O	0.66408700	0.41087400	1.84936700
W	-3.02333100	-0.76023400	-1.55739900
W	2.76398700	-2.06010700	-0.50845800
W	-0.26347800	-2.80871800	-2.05458700
O	-3.36156900	-0.76683000	-3.23870100
O	-4.48857900	-1.20550500	-0.78536600
O	2.94389400	-3.69014600	-0.00721500
O	4.22276500	-1.65608400	-1.31439400
O	-0.12971300	-4.45407300	-1.59090400
O	-0.54980800	-2.82209800	-3.74533300
O	2.96504700	-1.15302600	1.18722900
O	-2.00904300	-2.39365500	-1.33856800
O	1.57568600	-2.22064700	-2.02299800
O	1.94288800	0.21929200	-0.57761400
O	0.42166100	-1.98365100	0.13039200
O	-0.65197200	-0.41900600	-1.89437100
Pt	0.00600200	-0.00408000	-0.02248700
H	-0.33170700	0.19727200	-2.57218200
H	-0.09565100	2.37902000	-1.00026100
H	2.07057900	0.77192600	-1.36487900
H	-2.05856800	-0.78033600	1.31990000
H	0.34377900	-0.20530500	2.52724300
H	0.10759900	-2.38710600	0.95531300

$[H_6PtW_6O_{24}]^{1e/1H}$
-2 2

W	3.06900200	0.73186200	1.47224800
W	-2.73978200	2.04705900	0.62711200
W	0.33328000	2.77542600	1.97398100
O	3.32426600	0.73038100	3.17374100
O	4.58236200	1.19417400	0.80592700
O	-2.84094700	3.62481700	-0.04303800
O	-4.25066000	1.78893700	1.39298900
O	0.14276400	4.40086800	1.45887800
O	0.64290800	2.86380500	3.65903900
O	-3.08080900	1.19589000	-1.26048900
O	2.06333500	2.39187700	1.24323700
O	-1.56924900	2.21048300	2.09010800
O	-2.30063900	-0.14393700	0.48599500
O	-0.47556900	1.97739100	-0.11185800
O	0.68722500	0.44619700	1.86822500
W	-3.01921800	-0.89241900	-1.49761100
W	2.80569100	-2.02000100	-0.53136100
W	-0.19039600	-2.77749600	-2.04477600
O	-3.27702700	-0.71234200	-3.18571300
O	-4.54550300	-1.35544300	-0.87371600
O	2.89018900	-3.66560600	-0.03612300
O	4.30214000	-1.71615900	-1.31813700
O	-0.02059900	-4.42154500	-1.58762900
O	-0.52532200	-2.80169400	-3.72812500
O	3.03067300	-1.18603300	1.20183400
O	-2.01464900	-2.47369200	-1.31389100
O	1.62419000	-2.16511400	-2.07765000
O	2.19962800	0.20251500	-0.59836200
O	0.46213400	-2.00857000	0.08807800
O	-0.70339400	-0.46056400	-1.88015800
Pt	0.00315200	-0.01258700	-0.01006600
H	-0.38212600	0.14416100	-2.56461100
H	-0.18610800	2.40618200	-0.93049600
H	2.30483200	0.75702800	-1.38268000
H	-2.48360800	-0.69107600	1.26339500
H	0.32106000	-0.13705300	2.54878200
H	0.13069300	-2.42894100	0.89489300
H	-3.22478200	1.76340200	-2.02890800

$[H_6PtW_6O_{24}]^{2e/2H}$

-2 1

W	3.07488600	0.89640900	1.39512600
W	-2.81305500	2.00797800	0.66289300
W	0.24500200	2.75352500	1.97532200
O	3.25041300	0.64843200	3.09009700
O	4.62338400	1.46891800	0.93379600
O	-2.82136400	3.56450900	-0.07304400
O	-4.32917300	1.94584900	1.46241100
O	0.03716700	4.38199600	1.47787500
O	0.59681900	2.83359800	3.65303300
O	-3.14668400	1.19042000	-1.25171000
O	2.04152300	2.47916200	1.22304200
O	-1.62894300	2.17807000	2.13515400
O	-2.76064100	-0.16922800	0.59668600
O	-0.54533600	1.96366500	-0.06443400
O	0.74516400	0.48434700	1.84084500
W	-3.06581200	-0.90557900	-1.43626900
W	2.81683300	-2.00733300	-0.71975500
W	-0.25310000	-2.75362500	-2.00482000
O	-3.19824200	-0.69398600	-3.13970700
O	-4.62449600	-1.46383700	-0.99102200
O	2.84376900	-3.56842300	0.00600800
O	4.32460900	-1.93104400	-1.53385100
O	-0.04147300	-4.39117100	-1.53813600
O	-0.64549800	-2.79756500	-3.67447500
O	3.15889400	-1.19910400	1.19389700
O	-2.03784800	-2.48611200	-1.22474000
O	1.61812400	-2.17620200	-2.18090400
O	2.76731600	0.17485000	-0.64355100
O	0.55772600	-1.97880200	0.03633800
O	-0.73825600	-0.48952400	-1.84957500
Pt	0.00688500	-0.00643000	-0.00630500
H	-0.43607100	0.10424100	-2.55009000
H	-0.27667300	2.41189300	-0.87777200
H	3.36332900	0.58895500	-1.28112700
H	-3.33671300	-0.58949800	1.24812300
H	0.44320300	-0.11414200	2.53742000
H	0.30384600	-2.43178900	0.85171700
H	-3.11430500	1.75137400	-2.03766000
H	3.14112700	-1.76343000	1.97797600

H_2-from-$[H_6PtW_6O_{24}]^{2e/2H}$

W	2.99166000	0.81095700	1.48093200
W	-2.66901100	1.90143000	0.56431200
W	0.27817400	2.74689900	2.03705000
O	3.24359200	0.69233100	3.17015400
O	4.51841400	1.19201200	0.81000500
O	-2.93131600	3.52240300	0.04584300
O	-4.13802500	1.40246400	1.28937500
O	0.09662100	4.41602400	1.69435900
O	0.63960300	2.65057200	3.70924000
O	-3.04226500	1.26174100	-1.39256900
O	2.06055800	2.44356100	1.28501200
O	-1.54632700	2.11054200	2.08683700
O	-1.86074000	-0.05405800	0.18793100
O	-0.39119400	2.10478800	-0.15273200
O	0.66941600	0.42160600	1.67233900
W	-2.91536200	-0.83239700	-1.50303400
W	2.63553400	-2.12236800	-0.68167500
W	-0.17911300	-2.84544200	-2.05083300
O	-3.27149700	-0.73549800	-3.18749100
O	-4.39879200	-1.21687300	-0.73617500
O	2.85977200	-3.69763500	-0.03922800
O	4.16438000	-1.62279000	-1.29732100
O	-0.05691300	-4.48653500	-1.57165800
O	-0.56399400	-2.86051500	-3.72929000
O	2.93134900	-1.22934100	1.22601300
O	-1.94424100	-2.43506500	-1.32987900
O	1.73507500	-2.43351500	-2.30892700
O	2.12443900	0.19527100	-0.58277900
O	0.53264100	-1.85161500	-0.22151000
O	-0.58057200	-0.37776400	-2.10099600
Pt	0.07500100	0.08006000	-0.25510400
H	-0.31006300	0.25258500	-2.78479600
H	-0.12355600	2.61579700	-0.92971000
H	2.37857700	0.74245400	-1.34196700
H	-1.56085900	-1.41367000	2.21110300
H	0.28492500	-0.25529800	2.25292700
H	-1.14675000	-1.84855300	2.65677100
H	-3.60725600	1.76976400	-1.98896000
H	2.96567600	-1.79692200	2.00685100

$[H_6PtW_6O_{24}]^{3e/3H(O)}$

-2 2

W	3.13842300	0.95324500	1.57107400
W	-2.82984500	2.00267700	0.69983300
W	0.23391700	2.69433300	2.02981900
O	3.09742500	0.67157800	3.27186900
O	4.69202000	1.63011500	1.30371500
O	-2.82511100	3.57989000	0.00953400
O	-4.34687200	1.93197000	1.49744700
O	0.00751100	4.33802900	1.58986700
O	0.57417300	2.71200800	3.71120100
O	-3.17320400	1.24689300	-1.24108300
O	2.03818100	2.48126800	1.30265000
O	-1.64588100	2.10792400	2.16886800
O	-2.80936200	-0.17768000	0.56618300
O	-0.55689800	1.96066700	-0.04367600
O	0.73570700	0.43533700	1.79919800
W	-3.15799600	-0.83634000	-1.47504600
W	2.89786700	-1.91729400	-0.52129500
W	-0.40360600	-2.82035700	-1.96592900
O	-3.28458800	-0.57507200	-3.17193500
O	-4.73443800	-1.35152400	-1.04614600
O	2.65555400	-3.50376900	0.20171100
O	4.30318100	-1.89939500	-1.53313700
O	-0.31070300	-4.46285600	-1.48894700
O	-0.66116000	-2.85542500	-3.66140300
O	3.53002300	-1.10361900	1.30242200
O	-2.19373200	-2.47052200	-1.28805000
O	1.58548300	-2.40555500	-2.21546300
O	2.76635500	0.27596900	-0.39715100
O	0.48477600	-1.99279400	-0.07785700
O	-0.81065300	-0.50980700	-1.88174400
Pt	-0.03861400	-0.01802500	-0.04938800
H	-0.50459300	0.06852300	-2.59311000
H	-0.30436000	2.42814000	-0.85114400
H	2.27554500	0.84072500	-1.00514400
H	-3.38611000	-0.60642900	1.21180500
H	0.41395600	-0.15857800	2.49095000
H	0.28286600	-2.48919500	0.72845000
H	-3.14257000	1.82797600	-2.01228500
H	3.68572600	-1.66104100	2.07435100
H	1.94242700	-2.42660300	-3.11181700

$[H_6PtW_6O_{24}]^{3e/3H(Pt)}$
-2 2

W	2.93982000	0.86899600	1.50144700
W	-2.88145500	1.99255100	0.74714200
W	0.21832100	2.84032500	1.93929900
O	2.94758200	0.66207700	3.20905400
O	4.55717600	1.33416200	1.16169600
O	-3.31331000	3.56087800	0.19402000
O	-4.26316000	1.48041600	1.62882200
O	0.06495800	4.50835700	1.55676400
O	0.57818700	2.81562500	3.61688500
O	-3.27907100	1.13828600	-1.13735900
O	2.02779700	2.51933400	1.21502000
O	-1.65815500	2.24332800	2.16491100
O	-2.27380100	-0.18602000	0.51851200
O	-0.76133200	2.35628800	-0.10779800
O	0.62301300	0.60258700	1.64029100
W	-3.14204300	-0.95440900	-1.39366200
W	2.66648000	-2.13698500	-0.53448400
W	-0.29366800	-2.79208200	-2.05299000
O	-3.61145300	-0.79412300	-3.03920100
O	-4.57257600	-1.47519700	-0.60448900
O	2.61575100	-3.68018300	0.22101400
O	4.23380600	-2.12041900	-1.23556600
O	-0.16556900	-4.46284200	-1.69238900
O	-0.60457300	-2.74476000	-3.74417300
O	2.87868000	-1.25060000	1.36152200
O	-2.10157500	-2.52757600	-1.29812500
O	1.61077100	-2.27862000	-2.11554700
O	2.82093900	0.09444800	-0.54332900
O	0.38766300	-2.02114000	-0.06443400
O	-0.95263200	-0.47591000	-2.10969100
Pt	-0.16611500	0.20366400	-0.30617200
H	-0.78908900	0.08895400	-2.87497300
H	-0.60606500	2.88021600	-0.90289900
H	3.56229500	0.39047500	-1.08809200
H	-2.39296600	-0.74442000	1.29818800
H	0.25716600	-0.01857800	2.28475700
H	-0.00038300	-2.38183200	0.74351600
H	-3.53641300	1.69579700	-1.88263500
H	2.75583300	-1.77657800	2.16300200
H	1.14225000	0.56981400	-0.98197400

TS-3e-3H (HER)

-2 2

W	3.04230200	0.73666100	1.53616700
W	-2.86483100	1.98074400	0.73958500
W	0.31262000	2.78762100	1.90057500
O	2.85420300	0.51529200	3.24307800
O	4.66455400	1.26282000	1.35806400
O	-3.15462700	3.54778300	0.10242400
O	-4.27555100	1.61831000	1.64078300
O	0.13145200	4.40465600	1.35519400
O	0.66221500	2.90843300	3.57166900
O	-3.31476100	1.10422900	-1.12173000
O	2.07220500	2.37590400	1.19154300
O	-1.58269500	2.22098500	2.09714900
O	-2.33210400	-0.20433900	0.55650500
O	-0.68344000	2.12136700	-0.21383300
O	0.56651800	0.51746900	1.66299100
W	-3.13751800	-0.97492400	-1.40610100
W	2.77821600	-2.07213500	-0.47501300
W	-0.19640800	-2.70810200	-2.07621600
O	-3.56031000	-0.80162600	-3.06050400
O	-4.56396900	-1.55004800	-0.65305800
O	2.44513700	-3.58174600	0.30481600
O	4.33425400	-2.28643000	-1.16457500
O	-0.02339500	-4.36986500	-1.70437100
O	-0.50727200	-2.64879900	-3.76330100
O	3.24120400	-1.37622300	1.47927500
O	-2.02410800	-2.49118800	-1.32718300
O	1.65471300	-2.13013700	-2.04735400
O	2.72296500	-0.05476000	-0.30601500
O	0.33641500	-1.82013900	-0.02784400
O	-0.92330200	-0.37619700	-2.02153200
Pt	-0.13734700	0.15784200	-0.22023900
H	-0.73402000	0.24337700	-2.74090700
H	-0.49975300	2.59169000	-1.03986200
H	1.86216500	0.63197400	-1.14328600
H	-2.47633600	-0.75771900	1.33792600
H	0.12063600	-0.03114800	2.32566000
H	-0.10000200	-2.21914100	0.74004300
H	-3.54825400	1.66822000	-1.87082000
H	3.15553600	-1.92784400	2.26673700
H	1.20577700	1.01143700	-1.57056100

H_2-from-$[H_6PtW_6O_{24}]^{3e/3H(Pt)}$
-2 2

W	2.89830800	0.84566800	1.45035500
W	-2.91685100	2.02898400	0.70101400
W	0.09547100	2.73784500	2.13217300
O	3.15333100	0.65142900	3.15065300
O	4.40522100	1.39892900	0.84475100
O	-2.96610500	3.61624000	0.04685800
O	-4.45447100	1.79477900	1.41974400
O	-0.12059900	4.37861900	1.68235800
O	0.41944200	2.76264300	3.81682600
O	-3.21458500	1.21519700	-1.20990800
O	1.84107400	2.44435400	1.36011400
O	-1.79495400	2.16057500	2.20923700
O	-2.49630100	-0.14522000	0.54350900
O	-0.62434300	1.94760800	0.04221400
O	0.51673800	0.41986400	2.00471600
W	-3.16694800	-0.86989500	-1.45016400
W	2.66206100	-1.89237800	-0.60833100
W	-0.38535500	-2.76194900	-1.97576000
O	-3.34394900	-0.69971300	-3.14953800
O	-4.72164300	-1.31690200	-0.88841300
O	2.80818000	-3.49020600	0.03887200
O	4.13530100	-1.62525900	-1.44578800
O	-0.20266100	-4.41624900	-1.55955300
O	-0.76186700	-2.74827600	-3.64933700
O	3.18434500	-1.23376400	1.32197200
O	-2.19375700	-2.46811000	-1.22929300
O	1.43875500	-2.13478000	-2.06615800
O	2.08878000	0.04960800	-0.26257300
O	0.30049400	-2.04865600	0.15995400
O	-0.83329100	-0.46923800	-1.75983800
Pt	-0.13658700	-0.05067300	0.12674100
H	-0.46237400	0.11930700	-2.43364900
H	-0.28257300	2.38589400	-0.75090800
H	1.72476900	1.07770300	-2.35728500
H	-2.69099300	-0.70035100	1.31184300
H	0.18109800	-0.17504300	2.68980100
H	-0.01526900	-2.49907900	0.95592700
H	-3.31470600	1.79408500	-1.97660100
H	3.60036800	-1.77884300	2.00048700
H	1.44380800	1.39466900	-2.97514100

$[H_6PtW_6O_{24}]^{4e/4H(O)}$

-2 3

W	2.31291400	2.55807700	-0.24854800
W	-3.50168900	0.69954000	0.06360000
W	-1.26547200	3.23177600	0.18303200
O	2.47113800	3.71528900	1.05310300
O	3.16770700	3.09558600	-1.66247100
O	-4.16017200	1.48886300	-1.31708800
O	-4.86620100	0.44859400	1.06994400
O	-2.18927600	4.08830200	-0.97770700
O	-1.02739400	4.33327700	1.47457400
O	-3.32911900	-1.02522900	-1.06763600
O	0.45498500	3.48308800	-0.89770100
O	-2.61432800	2.11637300	1.00472700
O	-2.38646600	-0.87436700	1.04619300
O	-1.38974100	1.20313000	-0.92355500
O	0.42281900	1.87762800	0.96887600
W	-2.37492200	-2.69974700	-0.13270500
W	3.70167000	-0.71629700	0.07802600
W	0.98440800	-3.21194400	0.12978000
O	-2.38122000	-3.45337700	-1.69021300
O	-3.70548700	-3.39962200	0.70730900
O	4.18234600	-1.12332500	1.68502100
O	5.13933400	-0.75505600	-0.85404400
O	1.77701800	-3.80510800	1.56695500
O	0.98459000	-4.33320500	-1.20545200
O	3.66265300	1.26279800	0.72552600
O	-0.92150000	-3.42653500	0.75918500
O	2.96066100	-2.43569000	-0.61955400
O	2.56052100	0.43329200	-1.22058600
O	1.52809400	-1.04300500	0.91128400
O	-0.30958100	-1.66867400	-0.94791200
Pt	0.06537100	0.09047600	0.01805300
H	-0.20278400	-1.59499900	-1.90611600
H	-1.28097300	1.18839000	-1.88439000
H	2.96905200	0.55480400	-2.08831900
H	-2.52855600	-0.98566000	1.99428800
H	0.50473800	1.81597600	1.93129400
H	1.51362000	-0.99752100	1.87818100
H	-3.53098700	-1.02108200	-2.01125500
H	3.98247800	1.47882300	1.61117400
H	3.39220600	-2.88227200	-1.35886100
H	0.41996900	4.01818300	-1.70001500

TS-H-transfer-O-to Pt
-2 3

W	-2.87946200	0.92519200	0.00009900
W	-9.12831300	1.96743700	-0.68877800
W	-6.24753200	3.34551500	0.34617900
O	-3.18754800	0.83299000	1.69072400
O	-1.17657100	0.74431600	-0.12413300
O	-9.71873800	3.52338000	-1.14983100
O	-10.42731800	1.29048500	0.21526200
O	-6.59775700	4.99579600	-0.05302500
O	-5.29048500	3.33628800	1.78781200
O	-9.51669100	1.21780600	-2.61260600
O	-2.98575900	2.76835500	-0.48953000
O	-7.87542300	2.25044400	0.68168900
O	-8.34649100	-0.08891700	-1.04168800
O	-7.09727400	2.68936800	-1.63804600
O	-5.16655500	1.32000600	-0.19279500
W	-9.19767400	-0.83939200	-2.89786900
W	-3.44759300	-2.00520300	-1.97459200
W	-6.30475800	-2.50082500	-3.62677300
O	-9.67225000	-0.65387500	-4.54479700
O	-10.59511500	-1.47302900	-2.12633100
O	-3.69293800	-3.56965200	-1.30774900
O	-1.81001900	-2.08275900	-2.50240400
O	-6.10153800	-4.18213900	-3.36140200
O	-6.55935300	-2.35244800	-5.32511200
O	-3.39913900	-1.14882900	-0.09072900
O	-8.10084400	-2.38778700	-2.87346600
O	-4.35901600	-1.95888000	-3.67140300
O	-3.12182800	0.20996600	-2.01353700
O	-5.67228300	-1.65213700	-1.71349000
O	-7.07342700	-0.21753400	-3.64258200
Pt	-6.05813400	0.71489300	-2.07102500
H	-7.07128300	0.22692300	-4.49957200
H	-7.14464000	3.33878600	-2.34843500
H	-2.26044000	0.32753500	-2.43927300
H	-8.51218900	-0.66133000	-0.28097000
H	-5.55470900	0.69391600	0.43977400
H	-6.16791000	-1.87945900	-0.91642600
H	-9.85426300	1.77709300	-3.32235400
H	-4.21591300	-0.33017500	-4.37989400
H	-2.19286500	3.14566600	-0.89087200
H	-3.61282600	-1.65571900	0.70287400

O	-4.39078000	0.64446300	-4.48639500
H	-3.53119100	1.09240400	-4.51741500
H	-4.91828100	0.89826200	-3.37518100

$[H_6PtW_6O_{24}]^{4e/4H(Pt)}$

-2 3

W	0.79208200	-3.39733400	-0.06354200
W	-3.39753200	1.04149200	-0.11333400
W	-2.70115700	-2.30045500	-0.03701000
O	0.12520100	-4.09298700	-1.53062000
O	1.38999000	-4.62497700	1.00658700
O	-4.60676800	1.00166800	1.10542600
O	-4.13141200	1.93313600	-1.38148800
O	-3.90735800	-2.69474800	1.11720300
O	-3.03241300	-3.33040800	-1.36516600
O	-2.33116000	2.57455900	0.84562500
O	-1.23842300	-3.39388500	0.88116800
O	-3.37651700	-0.67979900	-0.91091600
O	-1.35108100	1.45776900	-0.96609800
O	-2.18267300	-0.43071500	1.22545400
O	-0.64768600	-1.65210000	-0.68045000
W	-0.63100500	3.39297600	-0.12092800
W	3.47989100	-1.00519600	-0.06933800
W	2.62318900	2.24905200	0.00247400
O	-0.41504100	4.52473100	1.15519300
O	-1.39703800	4.29854300	-1.36039600
O	4.25624200	-0.70525300	-1.58051800
O	4.68853500	-1.80467300	0.85493700
O	3.74234000	2.57810300	-1.25456700
O	2.99725100	3.39321800	1.23150800
O	2.46766100	-2.60585600	-1.03532300
O	1.06860500	3.12096000	-0.87561600
O	3.46915100	0.68846800	0.82342800
O	1.95188500	-1.87994000	1.16673100
O	1.75721500	0.36064900	-0.85505400
O	0.59007000	1.88449300	1.23854100
Pt	-0.13718600	0.09253800	0.45082900
H	0.55314700	1.97338000	2.19875800
H	-2.28489900	-0.40970000	2.18435700
H	2.31783200	-2.29432600	1.95852200
H	-1.24464100	1.34821800	-1.92053100
H	-0.60060700	-1.47538600	-1.63209400
H	1.52615900	0.36639900	-1.79327200
H	-2.63373700	2.93422300	1.68914200
H	2.64083000	-2.78096900	-1.96872300
H	0.59871200	-0.72836500	1.49308800
H	-1.50953800	-4.07872300	1.50557300

TS-4e-4H (HER)
-2 3

W	3.08941600	0.63849100	1.60913300
W	-2.77106900	1.98585800	0.67741100
W	0.23184400	2.77686800	2.06340800
O	2.90808600	0.35396500	3.30636500
O	4.65563400	1.32347800	1.48607900
O	-3.24219300	3.54127300	0.12538200
O	-4.14145900	1.41923200	1.53698800
O	0.27811100	4.45145100	1.67786000
O	0.62346600	2.71838800	3.72947000
O	-3.13534500	1.12386200	-1.19774400
O	2.19817700	2.51322100	1.29636700
O	-1.61933000	2.27579800	2.19752100
O	-1.96585700	-0.07795200	0.42997400
O	-0.65493600	2.47212900	-0.06800900
O	0.79053000	0.72049100	1.65949800
W	-2.94899000	-0.98896000	-1.39393000
W	2.85956100	-2.06918400	-0.52876600
W	-0.07918300	-2.76059400	-2.02827900
O	-3.56317700	-0.94280900	-2.99949600
O	-4.30135200	-1.47334300	-0.45029200
O	2.67083400	-3.60503200	0.24329700
O	4.42459600	-2.17273000	-1.23458600
O	0.10175200	-4.39157600	-1.52650200
O	-0.39806100	-2.87390300	-3.71831700
O	3.35334600	-1.40415500	1.48019900
O	-1.90283000	-2.54573000	-1.25205100
O	1.80115600	-2.20436900	-2.12473100
O	2.86233300	-0.02366600	-0.26694000
O	0.55084200	-1.79520700	-0.11876300
O	-0.87562300	-0.54641800	-2.27710100
Pt	0.09829900	0.42960600	-0.57022200
H	-0.80433100	-0.10768900	-3.13300900
H	-0.54172400	3.16250100	-0.73207400
H	2.03735500	0.67102100	-1.10344600
H	-2.00362500	-0.62440600	1.22703900
H	0.40940000	0.08522400	2.28189200
H	0.11328200	-2.09944400	0.68630700
H	-3.49244500	1.65492900	-1.92080000
H	1.43505200	1.10304300	-1.64675500
H	2.75047800	3.28835000	1.12908200
H	3.30104300	-1.96636500	2.26397000

H_2-S3-from-$[H_6PtW_6O_{24}]^{4e/4H(Pt)}$

-2 3

W	2.96165800	0.67141800	1.48428100
W	-2.77972800	2.08701700	0.52419200
W	0.08932000	2.83187900	2.09262600
O	3.25080300	0.47393300	3.17989300
O	4.42210600	1.33160100	0.87696000
O	-2.93473600	3.64414300	-0.18022800
O	-4.31713000	1.77561000	1.21695200
O	0.13623500	4.44308200	1.49831900
O	0.39914600	2.98912200	3.77350100
O	-3.02006800	1.19663100	-1.34753000
O	2.03335700	2.53244300	1.33332600
O	-1.78044600	2.38415000	2.14909700
O	-2.31860900	-0.09724700	0.48899400
O	-0.55067600	2.07246300	0.05372400
O	0.73964600	0.73055200	2.14721600
W	-3.00566100	-0.91641400	-1.51287600
W	2.70824100	-1.96433300	-0.67688800
W	-0.27628300	-2.81851400	-2.06928200
O	-3.39866100	-0.78736000	-3.18040100
O	-4.50244000	-1.30228300	-0.75828100
O	2.99276700	-3.57466600	-0.11338800
O	4.16699200	-1.55292600	-1.48994300
O	-0.09220900	-4.48751700	-1.69547300
O	-0.61948100	-2.80766000	-3.75579300
O	3.29075700	-1.35527500	1.31231900
O	-2.10151300	-2.56616600	-1.32703000
O	1.55958300	-2.17003100	-2.18601700
O	2.13344100	-0.03847000	-0.18826200
O	0.47469500	-2.28966000	0.03545200
O	-0.81088300	-0.59137400	-1.90243100
Pt	-0.05139500	-0.13065300	0.17780600
H	-0.46094800	0.04966200	-2.53358600
H	-0.20447600	2.48607400	-0.74679200
H	1.66310600	1.20633700	-2.19680900
H	-2.63255900	-0.60053400	1.25281700
H	0.57190700	0.26512100	2.97772300
H	0.23904500	-2.84318100	0.78841200
H	-3.16939800	1.74288700	-2.12967400
H	3.76325900	-1.88505200	1.96620600
H	1.26334900	1.64284000	-2.65521700
H	2.48793200	3.26769700	0.90166800

H_2-S1-from-$[H_6PtW_6O_{24}]^{4e/4H(Pt)}$

-2 3

W	3.11247200	0.60469000	1.42402100
W	-2.92409900	2.03972500	0.61606300
W	0.11004500	2.70997800	2.00931700
O	2.97672600	0.20848700	3.10854400
O	4.60122200	1.43555300	1.25972100
O	-2.93197100	3.56859200	-0.17320100
O	-4.44381100	2.00351800	1.40315300
O	0.14187000	4.34115300	1.47786400
O	0.51046300	2.76261900	3.67312300
O	-3.22891900	1.14760500	-1.24264700
O	2.04403300	2.41223200	1.22977000
O	-1.75715600	2.29126900	2.13081500
O	-2.83829400	-0.12600100	0.67828100
O	-0.65087000	2.00972100	-0.07323600
O	0.65924600	0.58448500	1.78780500
W	-3.09761000	-0.96999900	-1.34931400
W	2.89144600	-1.98107700	-0.70171500
W	-0.21637000	-2.75588500	-1.94694600
O	-3.35421900	-0.79312200	-3.04255900
O	-4.61266300	-1.56767000	-0.80803700
O	2.81369000	-3.52383600	0.08334100
O	4.34451900	-2.02752200	-1.61702100
O	0.01152600	-4.38437200	-1.45512700
O	-0.61276700	-2.84301500	-3.61785300
O	3.80830600	-1.34893900	1.19212300
O	-2.02442200	-2.51040800	-1.15774000
O	1.61547800	-2.13720600	-2.12515100
O	2.65607900	-0.04226500	-0.33413200
O	0.49822500	-1.92130000	0.07074600
O	-0.81447500	-0.49395800	-1.88232200
Pt	-0.07845600	0.03942600	-0.05379600
H	-0.54560800	0.10825200	-2.58882700
H	-0.39954700	2.49910000	-0.86769200
H	2.29441300	1.73989300	-2.46048300
H	-3.48392500	-0.49735500	1.29437400
H	0.40190500	0.00202400	2.51736000
H	0.14570100	-2.35337800	0.86026600
H	-3.22490600	1.67821400	-2.05049300
H	3.81707200	-1.93147700	1.96225000
H	1.57322900	1.86890400	-2.59705200
H	2.53246700	3.19869000	0.95199300

Supplementary References

1. Zhang, J. *et al*. Single platinum atoms immobilized on an MXene as an efficient catalyst for the hydrogen evolution reaction. *Nat. Catal.* **1**, 985-992 (2018).

2. Zhang, H. *et al.* Dynamic traction of lattice-confined platinum atoms into mesoporous carbon matrix for hydrogen evolution reaction. *Sci. Adv.* **4**, eaao6657 (2018).

3. Cheng, N. *et al.* Platinum single-atom and cluster catalysis of the hydrogen evolution reaction. *Nat. Commun.* **7**, 13638 (2016).

4. Chen, W. *et al.* Single Tungsten atoms supported on MOF-derived N-doped carbon for robust electrochemical hydrogen evolution. *Adv. Mater*. 30, 1800396 (2018).

5. Qin, J-S. *et al.* Ultrastable polymolybdate-based metal−organic frameworks as highly active electrocatalysts for hydrogen generation from water. *J. Am. Chem. Soc.* **137**, 7169-7177 (2015).

6. Jing, S. *et al*. Carbon-encapsulated WO_x hybrids as efficient catalysts for hydrogen evolution. *Adv. Mater*. 30, 1705979 (2018).

7. Tang, K., Wang, X., Li, Q. & Yan, C. High edge selectivity of in situ electrochemical Pt deposition on edge-rich layered WS_2 nanosheets. *Adv. Mater.* **30**, 1704779, (2018).

8. Tavakkoli, M. *et al.* Electrochemical activation of single-walled carbon nanotubes with pseudo-atomic-scale platinum for the hydrogen evolution reaction. *ACS Catal.* **7**, 3121-3130 (2017).

9. Huang, X. *et al*. Solution-phase epitaxial growth of noble metal nanostructures on dispersible single-layer molybdenum disulfide nanosheets. *Nat. Commun.* **4**, 1444 (2013)

10. Zhu, L. *et al.* A rhodium/silicon co-electrocatalyst design concept to surpass platinum hydrogen evolution activity at high overpotentials. *Nat. Commun*. **7**, 12272 (2016).

11. Li, M. *et al.* Pt monolayer coating on complex network substrate with high catalytic activity for the hydrogen evolution reaction. *Sci. Adv*. **1**, e1400268 (2015).

12. J. N. Tiwari, *et al.* Multicomponent electrocatalyst with ultralow Pt loading and

high hydrogen evolution activity. *Nat. energy*. **3**, 773-782 (2018).

13. Deng, J. *et al*. Triggering the electrocatalytic hydrogen evolution activity of the inert two-dimensional MoS_2 surface via single-atom metal doping. *Energy Environ. Sci*. **8**, 1594-1601 (2015).

14. Chen, W. *et al*. Rational design of single molybdenum atoms anchored on N-doped carbon for effective hydrogen evolution reaction. *Angew. Chem*. **129**, 16302-16306 (2017).

15. Cao, L. *et al*. Identification of single-atom active sites in carbon-based cobalt catalysts during electrocatalytic hydrogen evolution. *Nat. Catal*. **2**, 134-141 (2019).

16. Chen, J. *et al*. Enhanced activity for Hydrogen Evolution Reaction over CoFe Catalysts by Alloying with small amount of Pt. *ACS Appl. Mater. Interfaces* **9**, 3596−3601 (2017).
